# Mitochondrial protein import receptors in Kinetoplastids reveal convergent evolution over large phylogenetic distances

**DOI:** 10.1038/ncomms7646

**Published:** 2015-03-26

**Authors:** Jan Mani, Silvia Desy, Moritz Niemann, Astrid Chanfon, Silke Oeljeklaus, Mascha Pusnik, Oliver Schmidt, Carolin Gerbeth, Chris Meisinger, Bettina Warscheid, André Schneider

**Affiliations:** 1 Department of Chemistry and Biochemistry, University of Bern, Freiestrasse 3, Bern CH-3012, Switzerland; 2 Department of Biochemistry and Functional Proteomics, Faculty of Biology and BIOSS Centre for Biological Signalling Studies, University of Freiburg, Freiburg 79104, Germany; 3 Institut für Biochemie und Molekularbiologie, ZBMZ and BIOSS Centre for Biological Signalling Studies, Universität Freiburg, Freiburg 79104, Germany

## Abstract

Mitochondrial protein import is essential for all eukaryotes and mediated by hetero-oligomeric protein translocases thought to be conserved within all eukaryotes. We have identified and analysed the function and architecture of the non-conventional outer membrane (OM) protein translocase in the early diverging eukaryote *Trypanosoma brucei*. It consists of six subunits that show no obvious homology to translocase components of other species. Two subunits are import receptors that have a unique topology and unique protein domains and thus evolved independently of the prototype receptors Tom20 and Tom70. Our study suggests that protein import receptors were recruited to the core of the OM translocase after the divergence of the major eukaryotic supergroups. Moreover, it links the evolutionary history of mitochondrial protein import receptors to the origin of the eukaryotic supergroups.

## Abstract (web summary)

Protein translocation into the mitochondrion is a conserved process in all eukaryotes. Here, Mani *et al.* describe a non-conventional translocase complex in *Trypanosoma brucei* and propose that protein import receptors were recruited to the core complex after the divergence of the major eukaryotic supergroups.

## Graphical abstract

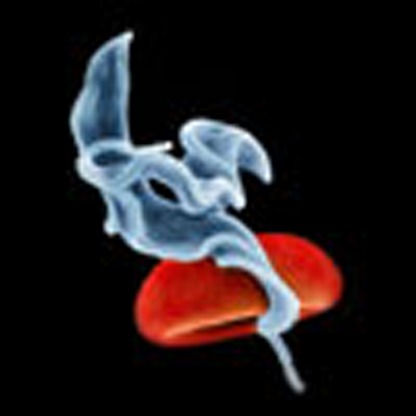

The origin of eukaryotes is tightly linked to a single endosymbiotic event between a probably prokaryotic host cell and an α-proteobacterium that subsequently was converted into a mitochondrion. At the heart of this organellogenesis lays the evolution of a protein import system^1^,^2^,^3^. Only the presence of such a system allowed the ancestor of the mitochondrion to profit from proteins whose genes it had transferred to the genome of the host cell. Today >95% of all mitochondrial proteins are imported into the organelle, a process that is mediated by translocases in the outer and inner membranes^4^,^5^. The hetero-oligomeric translocase of the outer membrane (TOM) is of special interest since it is at the interface between the organelle and the cytosol. Essentially all imported proteins irrespective of their final intramitochondrial localization require TOM to be translocated across the OM. TOM consists of (i) the pore-forming β-barrel protein Tom40, (ii) the TOM complex organizer Tom22 that also functions as a secondary receptor^6^, (iii) the three small proteins Tom5, Tom6 and Tom7, which function in the regulation of TOM complex assembly and (iv) the receptor subunits Tom20 and Tom70^7^. The latter two are signal-anchored proteins with a single tetratricopeptide repeat (TPR) in the case of Tom20 or 11 TPR motifs in the case of Tom70 and have in part overlapping functions. While Tom20 primarily binds to the presequence of precursor proteins, Tom70 has a preference to bind hydrophobic internal targeting sequences^8^,^9^ .

The machinery and the mechanism of mitochondrial protein import have been analysed in great detail. However, with the notable exception of plants^10^, these studies are phylogenetically biased, because they essentially have been performed only in fungi, which belong to the eukaryotic supergroup of the Opisthokonts. Bioinformatic analyses indicate that the core components Tom40 and Tom22 might be present in all eukaryotes^2^. However, whether and to which extent the other Tom subunits, including the Tom20 and Tom70 receptors, are conserved is unclear^11^,^12^.

The parasitic protozoan *Trypanosoma brucei*, a representative of the supergroup of the Excavates, is an excellent, experimentally highly accessible model system to identify and investigate diverged features of the mitochondrial protein import machinery^13^. In contrast to most other eukaryotes, bioinformatic analyses failed to identify any orthologues of TOM complex subunits in the trypanosomal genome^13^. Thus, it needed a biochemical approach to identify the import pore the only known component of the trypanosomal OM translocase^14^. It consists of a β-barrel protein that as Tom40 can be grouped into the mitochondrial porin protein family^15^,^16^,^17^. However, it also shows similarities to the Omp85-like protein family of bacterial protein translocases. A direct electrophysiological comparison of the recombinant trypanosomal protein with recombinant Tom40 showed that the former—unlike Tom40—shares physical features with Omp85-like plastid and bacterial pores^18^. Thus, the protein was initially termed archaic TOM (ATOM)^14^,^18^. To keep the nomenclature consistent with other systems such as yeast and plants, we decided to rename ATOM to ATOM40, the number indicating the approximate molecular weight of the protein. The term ATOM without a number will now stand for the entire ATOM complex including all of its subunits.

ATOM40 migrates in a high molecular weight complex of ~700 kDa when analysed by blue native polyacrylamide electrophoresis (BN–PAGE)^14^. In the present study we have identified and functionally analysed the ATOM complex subunits. In addition, we have delineated the architecture of the ATOM complex. We show that it consists of at least six subunits. Two of them are novel protein import receptors with overlapping substrate specificities that evolved independently from Tom70 and Tom20 of fungi and humans.

## Results

### Identification of ATOM complex subunits

**Figure 1:**
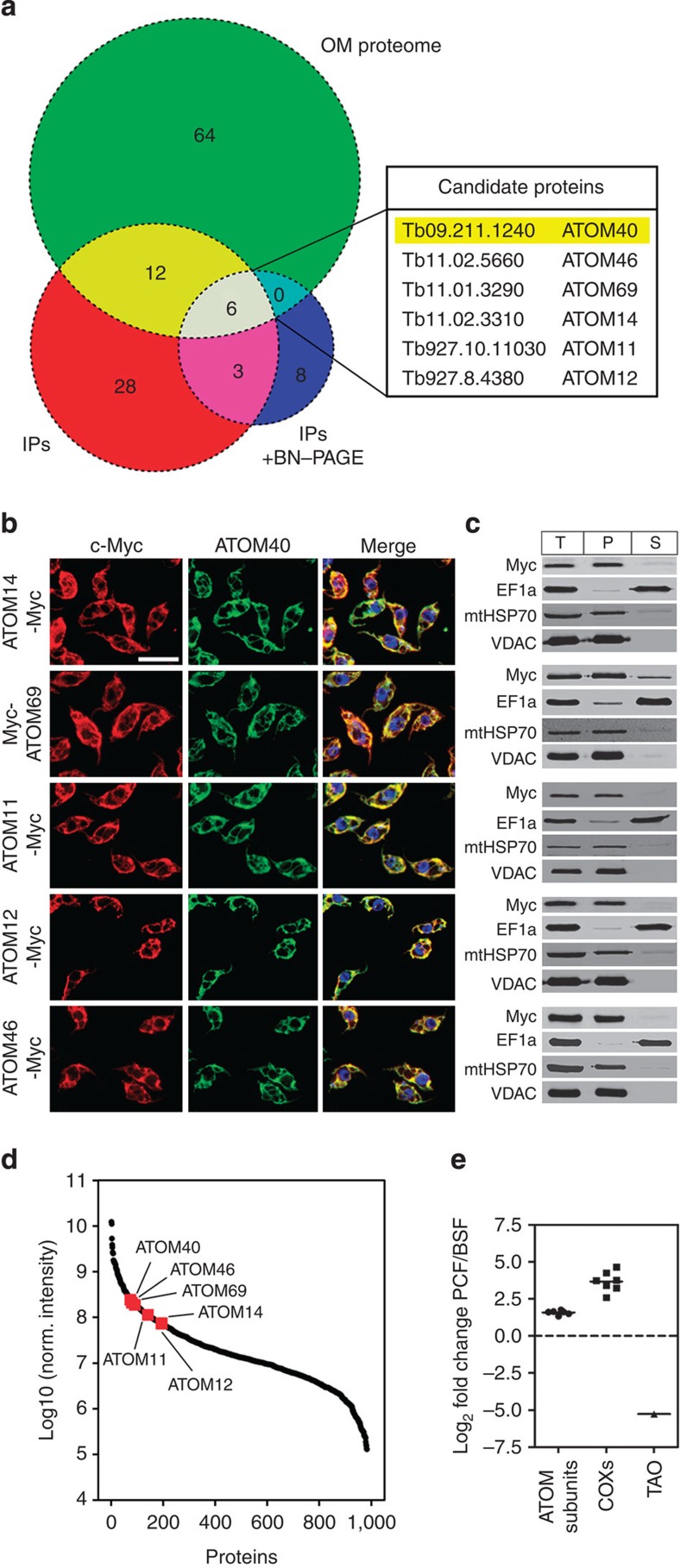
Identification of ATOM complex subunits. (**a**) Venn diagram showing the overlap of the *T. brucei* OM proteome (green)^19^ with proteins identified in IPs using mitochondria isolated from cells expressing HA-tagged ATOM40. Elution was either done under denaturing conditions (red) (Supplementary Data 1) or under native condition with subsequent size selection by BN–PAGE (blue) (Supplementary Data 2). (**b**) Immunofluorescence microscopy of c-Myc-tagged candidate proteins (red) and ATOM40 (green). Merge pictures include staining with 4′,6-diamidino-2-phenylindole (DAPI) to visualize nuclear and mitochondrial DNA (blue). Bar, 10 μm. (**c**) Immunoblot analysis of c-Myc-tagged candidate proteins in whole cells (T), crude mitochondrial (P) and cytosolic fractions (S). EF1a, mtHSP70 and VDAC served as cytosolic or mitochondrial marker proteins, respectively. (**d**) Relative abundance of the putative ATOM complex subunits (red) estimated by normalized intensity values of 1,056 proteins identified by mass spectrometry of gradient-purified mitochondria^19^. (**e**) Relative abundance differences between insect stage (PCF) and bloodstream form (BSF) *T. brucei* of putative ATOM complex subunits, subunits of the cytochrome *c* oxidase (COXs) and terminal alternative oxidase (TAO)^21^ (see also Supplementary Fig. 1).

Using a quantitative proteomics approach, we have recently shown that the trypanosomal mitochondrial OM proteome consists of 82 proteins^19^. To identify which of these proteins are candidates for ATOM complex subunits immunoprecipitations (IPs) were performed. Mitochondria were gradient-purified from cells expressing haemagglutinin (HA)-tagged ATOM40, solubilized by digitonin and subjected to IPs using anti-HA antibodies. The experiment was performed in duplicate and IPs using wild-type mitochondria lacking tagged ATOM40 served as controls. Mass spectrometric analysis identified 49 proteins that reproducibly and specifically co-purified with HA-tagged ATOM40 under denaturing elution conditions (Supplementary Data 1). Furthermore, a variation of the same IP experiment was done in which the elution was performed under native conditions using an excess of HA peptide. Subsequently, the protein complexes present in the eluates were separated by BN–PAGE. Gel lanes were cut into equal slices and analysed by mass spectrometry, which resulted in the identification of 17 proteins that were co-enriched with HA-tagged ATOM40 (Supplementary Data 2). The intersection of the three data sets (‘OM proteome’/‘IP’/‘IP+BN–PAGE’) contained ATOM40 and five additional proteins, which were considered prime candidates for ATOM complex subunits (Fig. 1a).

According to their predicted molecular weight the candidate proteins were termed ATOM69, ATOM46, ATOM14, ATOM12 and ATOM11. They are well conserved among Kinetoplastids (Supplementary Table 1). However, with the exception of ATOM14, which shows some limited similarity to Tom22, homology search programs such as (PSI)-BLAST or HHPred^20^ failed to identify homologous proteins in other organisms except for proteins that contain shared conserved domains (see below).

To verify that the five candidates indeed are ATOM complex subunits, we performed reciprocal IPs (Supplementary Fig. 1). To that end the five candidates were tagged at their N- and C-termini using the c-Myc epitope. In all cases, IPs of HA-tagged ATOM40 pulled down the c-Myc-tagged candidate proteins and *vice versa*. Except for ATOM46, where only the C-terminally tagged version was mitochondrially localized, it did not matter on which side the proteins were tagged. Furthermore, immunofluorescence analysis and digitonin-based enrichment of crude mitochondrial fractions indicated that all five candidates exclusively localize to mitochondria (Fig. 1b,c).

All ATOM complex subunit candidates show similar relative abundances in the mitochondrial proteome, as might be expected for proteins that form a hetero-oligomeric complex (Fig. 1d). Many mitochondrial proteins such as cytochrome *c* oxidase (Cox) and alternative oxidase (TAO) are stage specifically regulated^21^. Mitochondrial protein import, however, is constitutively active. In line with this all putative ATOM complex subunits showed similar and relatively minor changes in abundance between the two life cycle stages. The higher amounts of the proteins observed in the insect form is consistent with the larger size of the mitochondrion in this stage (Fig. 1e)^22^.

We also tested a number of the 12 proteins that were only present in the intersection of the two data sets ‘OM proteome’ and ‘IP’ (Fig. 1a and Supplementary Fig. 1). Neither of these proteins fulfilled all the criteria defined for ATOM complex subunits that are discussed above. Moreover, we recently described, pATOM36, an essential mitochondrial OM protein that is implicated in the import of a subset of mitochondrial proteins and loosely associated with ATOM40 (ref. 23). However, pATOM36 is not a *bona fide* subunit of the ATOM complex because it does neither routinely co-immunoprecipitate with ATOM40 nor does it co-migrate with the ATOM complex on BN–PAGE. In summary, we conclude that the ATOM complex consists of six subunits.

### Most ATOM complex subunits are essential

**Figure 2:**
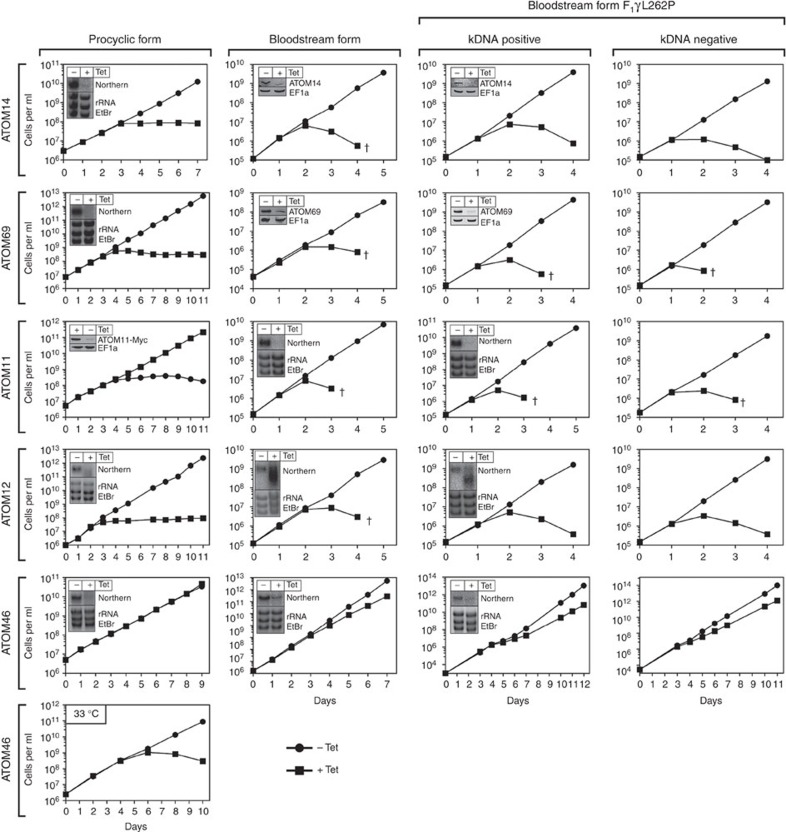
ATOM complex subunits are essential. Growth curves of uninduced (-Tet) and induced (+Tet) procyclic and bloodstream forms of the indicated knockdown cell lines. Procyclic cells were grown at 27 °C (and for ATOM46 also at 33 °C) and bloodstream forms at 37 °C. All experiments are based on tetracycline (Tet)-inducible RNAi cell lines, except for ATOM11 in procyclic cells for which a conditional knockout cell line was used. This cell line allows depletion of ATOM11 in the absence of Tet. F_1_γL262P is a bloodstream form cell line that can grow in the absence of the kDNA^26^. It has been tested in the presence of kDNA (positive) or after removal of the kDNA (negative) by treatment with 10 nM ethidiumbromide (Supplementary Fig. 3). Insets, Northern blots or immunoblots confirming successful knockdowns.

During its life cycle *T. brucei* alternates between the Tsetse fly and a mammalian host. This requires many adaptations some of which concern the mitochondrion. Insect-stage or procyclic trypanosomes have a highly active mitochondrion that can generate ATP by oxidative phosphorylation. The long slender bloodstream form present in the mammalian host, in contrast, has a smaller mitochondrion that lacks the respiratory complexes^24^. To examine the biological importance of the ATOM complex subunits during the life cycle, we produced inducible knockdown cell lines for both the procyclic and the bloodstream forms. The results in Fig. 2 show that all ATOM complex subunits, except ATOM46, are essential in both life cycle stages. Ablation of ATOM46 in contrast does not affect growth of insect-stage cells and only marginally slows down growth of bloodstream forms. However, the protein becomes essential for the insect-stage when grown at elevated temperature. The immunoblots in Supplementary Fig. 2 show that this is a direct effect of the ablation of ATOM46 since the levels of the other ATOM complex subunits are not affected. Thus, growth at 33 °C requires higher levels of ATOM46 than growth at 27 °C.

Trypanosomes require mitochondrial gene expression and thus mitochondrial DNA, termed kinetoplast DNA (kDNA), throughout their life cycle^25^. However, recently a bloodstream form cell line was engineered, termed F_1_γL262P, that due to a single compensatory mutation in the nuclear-encoded γ-subunit of the mitochondrial ATPase can grow in the absence of kDNA^26^. Interestingly, ablation of ATOM complex subunits in F_1_γL262P cells, results in the same phenotypes than observed in normal bloodstream form cells (Fig. 2), even when the mitochondrial genome had been removed by ethidiumbromide treatment (Fig. 2 and Supplementary Fig. 3). These results demonstrate that the ATOM complex subunits, consistent with their proposed role in protein import, perform a more fundamental function than simply maintaining mitochondrial gene expression.

Moreover, the results in the F_1_γL262P cells allow for a direct comparison with *Saccharomyces cerevisiae* which can also grow in the absence of mitochondrial DNA. This comparison shows that in trypanosomes all but one of the six ATOM complex subunits are essential for viability, whereas in yeast the only essential subunit of the TOM complex is the pore-forming Tom40 (ref. 7).

### ATOM complex subunits are required for protein import

**Figure 3:**
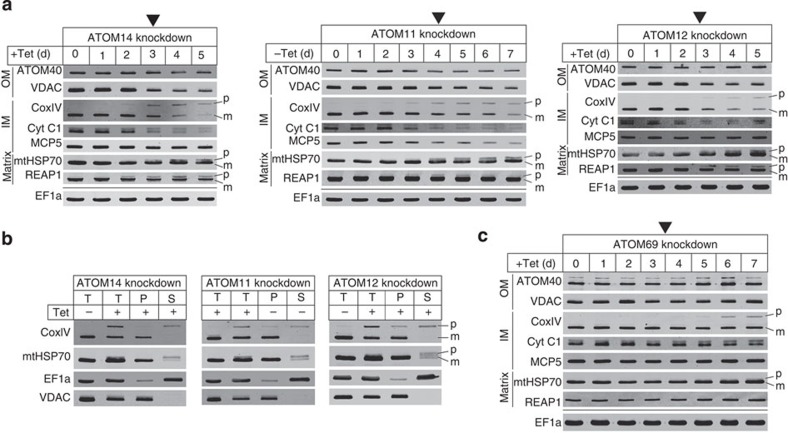
*In vivo* protein import defects. (**a**) Immunoblots showing the steady-state levels of ATOM40, VDAC, CoxIV, cytochrome *c*1 (Cyt *c*1), MCP5, mtHSP70 and REAP1 in whole-cell extracts of the indicated knockdown cell lines. Cytosolic EF1a serves as a control. Time of induction in days (d) is indicated at the top. Black triangles indicate the onset of the growth phenotype. The position of precursor (p) and mature forms (m) are indicated. (**b**) Immunoblot analysis of whole-cell (T), digitonin-extracted, mitochondria-enriched pellet (P) and soluble (S) fractions of the indicated knockdown cells. VDAC and EF1a serve as markers for mitochondria and cytosol, respectively. (**c**) As in **a** but results are for ATOM69.

To measure the effects ablation of each of the four essential ATOM subunits has on *in vivo* import of mitochondrial proteins, we prepared protein extracts of the corresponding inducible knockdown cell lines. The extracts were collected at the indicated times after induction and analysed on immunoblots using a panel of antibodies recognizing imported mitochondrial proteins (Fig. 3a). We have previously shown that, depending on the imported substrates, *in vivo* ablation of protein import factors causes cytosolic accumulation of unprocessed precursor proteins and/or a decrease of the mitochondrial localized mature forms. However, accumulation of precursors is only seen for some substrates because mislocalized precursor proteins that accumulate in the cytosol are often rapidly degraded^14^,^23^.

Figure 3a shows that knockdown of ATOM14, ATOM11 and ATOM12 causes an accumulation of precursors for Cox subunit IV (CoxIV), mitochondrial heat shock protein 70 (mtHsp70) and RNA editing-associated protein 1 (REAP1) that becomes apparent approximately at the onset of the growth arrest. As expected due to the lack of import these unprocessed precursors localize to the cytosolic fraction (Fig. 3b). Moreover, a reduction in the levels of essentially all tested mitochondrial proteins is also seen, whereas the level of cytosolic elongation factor 1a (EF1a) that serves as a control for a non-imported protein is not affected. The decrease in the levels of ATOM40 and the mitochondrial carrier protein 5 (MCP5) are strongest in ATOM11, more moderate in ATOM14 and not seen in ATOM12 knockdown cell lines. Ablation of ATOM69, on the other hand, causes precursor accumulation for CoxIV and possibly mtHsp70 only (Fig. 3c). It appears 2 days after the onset of the growth arrest and the steady-state levels of mitochondrial proteins are only slightly affected. In summary, these experiments show that the individual ablation of all four essential ATOM complex subunits affects *in vivo* import of mitochondrial proteins to various degrees.

**Figure 4:**
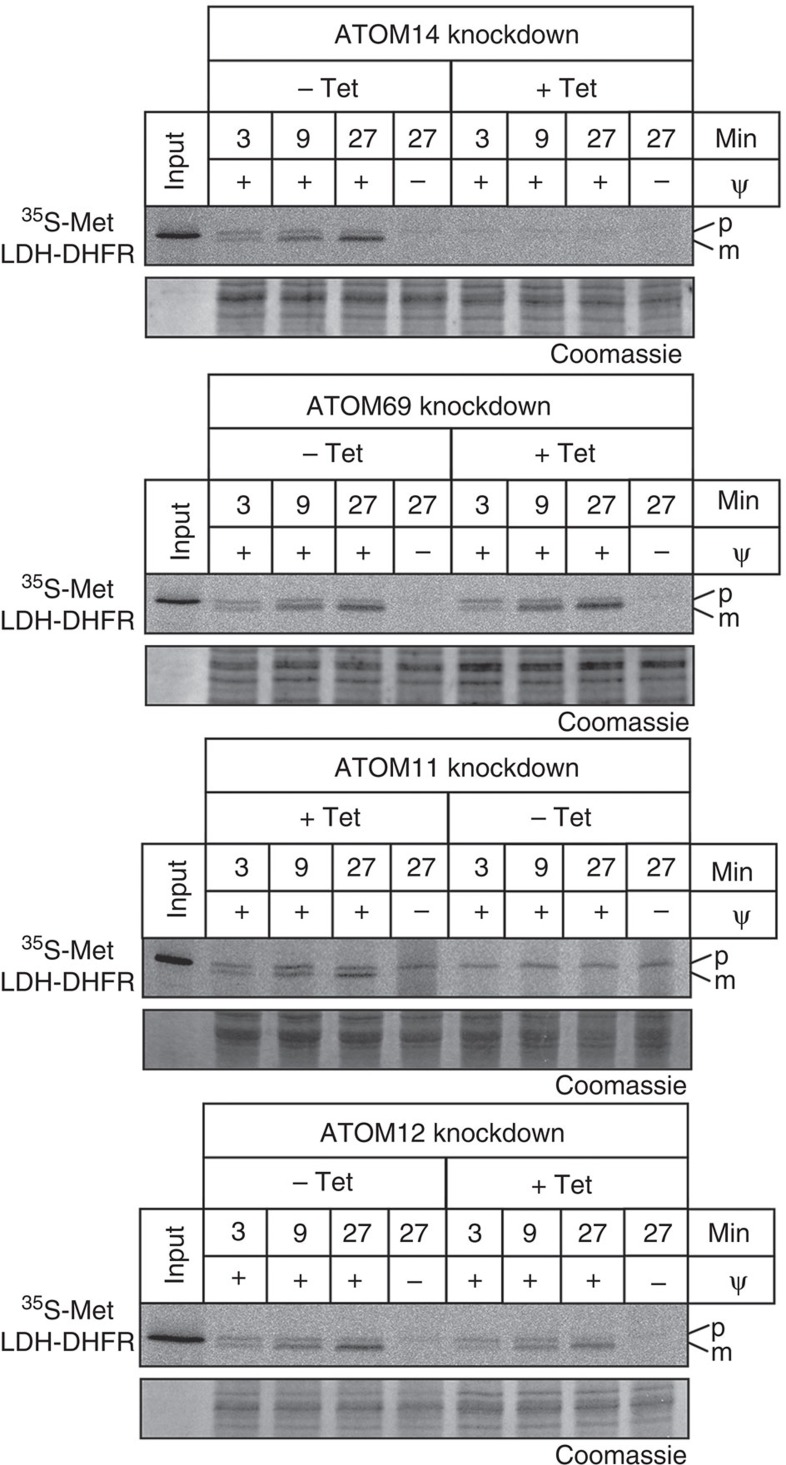
*In vitro* protein import defects. ^35^S-Met-labelled LDH–DHFR was imported into mitochondria isolated from the indicated uninduced and induced knockdown cell lines. All import reactions were treated with proteinase K and analysed by SDS–PAGE followed by autoradiography. Ψ, membrane potential. Input, 10% of the added substrate, Coomassie-stained gels are shown as loading controls. The position of precursor (p) and mature forms (m) are indicated.

Mitochondrial protein import was also assayed *in vitro*^27^ using mitochondria isolated from uninduced and induced knockdown cell lines. To prevent pleiotropic effects, mitochondria from induced cells were isolated prior to the onset of the growth arrest. Figure 4 shows that import of the *in vitro* translated chimeric protein consisting of the presequence-containing 150 N-terminal amino acids of lipoamide dehydrogenase (LDH) fused to mouse dihydrofolate reductase (DHFR) was essentially abolished in mitochondria isolated from ATOM11- and ATOM14-ablated cells and significantly reduced in mitochondria lacking ATOM12. Interestingly, import into mitochondria ablated for ATOM69 was not affected.

### ATOM complex architecture

**Figure 5:**
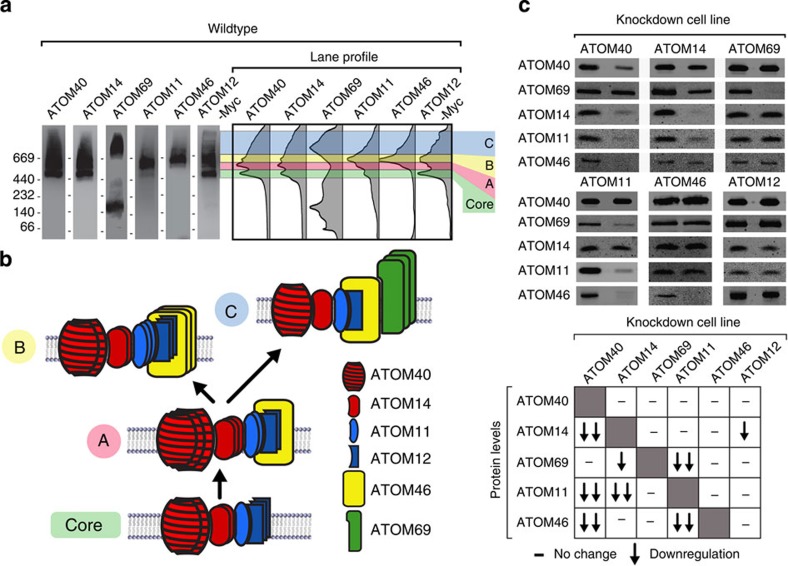
ATOM complex architecture and functional interactions between its subunits. (**a**) BN–PAGE immunoblots of mitochondrial membrane extracts from wild-type cells were probed with antisera against the indicated subunits. For ATOM12, a c-Myc tag version of the protein was analysed. Quantifications of lane profiles revealed the presence of four high molecular weight complexes termed: core, A, B and C. Molecular weight markers (kDa) are indicated. (**b**) Model of the composition of the complexes. Arrows indicate the suggested assembly pathway. (**c**) Top panels, SDS–PAGE immunoblot analysis of steady-state levels of individual ATOM complex subunits in all knockdown cell lines. Left and right lanes represent uninduced and induced cell lines, respectively. Lower panel, summary of the immunoblotting data (see also Supplementary Fig. 4).

BN–PAGE has been used extensively to study large membrane protein complexes. We applied this method in combination with immunoblots to digitonin-solubilized mitochondrial membranes of wild-type *T. brucei* cells using antisera against ATOM complex subunits (Fig. 5a). BN–PAGE resolves four ATOM40-containing protein complexes, whose molecular masses range from 450 to 1000, kDa and whose composition is illustrated in the model shown in Fig. 5b. The smallest of them is the ATOM core complex. It consists of ATOM40 and ATOM14, which show essentially identical profiles on BN gels (Fig. 5a), but also contains ATOM12 and some ATOM11. Because no ATOM12 antiserum was available, ATOM12 could only be detected in a cell line expressing the tagged protein. Complex A is identical to the core complex but contains higher amounts of ATOM11 and some ATOM46. Complex B is based on complex A but contains larger amounts of ATOM11 and especially of ATOM46. The main component of complex C, the largest of the four complexes, is ATOM69. However, based on the results described below, lower amounts of all the other subunits also contribute to complex C.

### Ablation of individual subunits differentially affects ATOM

To reveal the functional connections between the ATOM complex subunits, we measured how their ablation affects the abundance of the other subunits as detected by SDS–PAGE immunoblotting (Fig. 5c). To minimize indirect effects, the knockdown cell lines were analysed prior to the onset of the growth arrest.

The results illustrate the central role of ATOM40 for the stability of the whole complex because in its absence ATOM14, ATOM46 and ATOM11 are also strongly depleted. ATOM14 is required for the stability of ATOM11 and to a limited extent of ATOM69. ATOM69 and ATOM46 appear to be more peripheral components because their absence does not influence any other subunits. ATOM11, on the other hand, has a specific and strong influence on ATOM69 and ATOM46, which are unstable in its absence, suggesting that it mediates their association with the ATOM core complex. The absence of ATOM12 finally leads to a slight decrease of ATOM14 only.

We also analysed by BN–PAGE how the ablation of individual ATOM complex subunits affects the composition of the four complexes (Supplementary Fig. 4). This analysis confirms the results of Fig. 5a and supports the model shown in Fig. 5b. The BN–PAGE analysis of the knockdown cell lines in addition shows that:

(i) Interaction of ATOM69 with the core complex requires at least a small amount of ATOM46 (Supplementary Fig. 4e). ATOM46, however, can interact with the core complex in the absence of ATOM69 (Supplementary Fig. 4c). These results suggest an ordered assembly pathway of the four complexes as indicated by the arrows in Fig. 5b.

(ii) ATOM12, which is mainly found in the core complex and in complex B, may have an antagonistic role to ATOM11. In its absence much of the ATOM core complex is shifted into the largest complex C. ATOM12 therefore appears to prevent interaction of ATOM69 with complex A and thus the formation of complex C (Supplementary Fig. 4f).

(iii) With the exception of ATOM46, all ATOM complex subunits are essential under all growth conditions (Fig. 2). Of all four complexes, however, only the ATOM core complex appears to be essential. This is illustrated by the fact that while ATOM69 is an essential protein it does not need to be integrated into complex C to be functional (Supplementary Fig. 4e).

### Domain structure and topology of ATOM complex subunits

**Figure 6:**
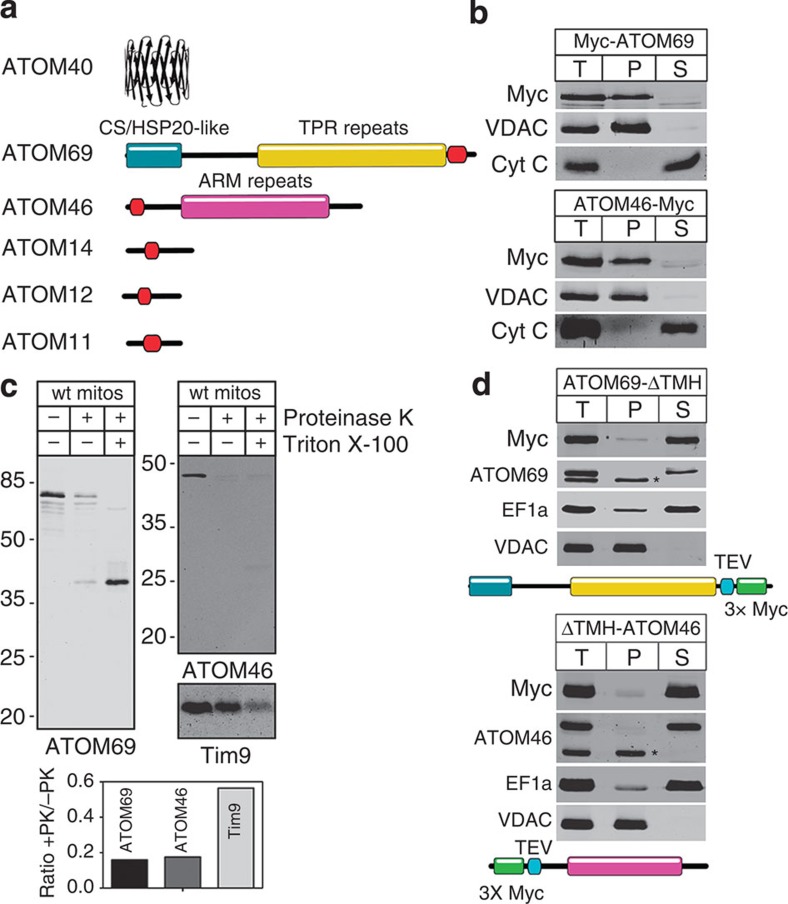
Domain structure and topology of ATOM complex subunits. (**a**) Predicted domain structure of ATOM complex subunits drawn to scale. The predicted domains are indicated in colours. Red, predicted transmembrane domains. (**b**) Immunoblots of the total (T), pellet (P) and supernatant (S) fractions of carbonate-extracted mitochondria isolated from cells expressing c-Myc-tagged ATOM69 and ATOM46 performed at pH 11.5 and analysed by anti-c-Myc antiserum. VDAC and cytochrome *c* (Cyt *c*) serve as marker for an integral and peripheral membrane protein, respectively (**c**). Immunoblots of a protease protection assay probed for ATOM69 and ATOM46 using gradient-purified wild-type mitochondria. The intermembrane space protein Tim9 serves as a control. Bottom graph, quantification of the ratios between untreated and proteinase K-treated samples of the indicated proteins. (**d**) Immunoblot analysis of subcellular fractions from transgenic trypansomes expressing c-Myc-tagged variants of ATOM69 and ATOM46 that lack the predicted transmembrane domains. Whole cells (T), digitonin-extracted crude mitochondria (P) and cytosol (S) were analysed. VDAC and EF1a serve as mitochondrial or cytosolic markers, respectively. TEV, TEV protease cleavage site. Asterisks indicate the untagged versions of ATOM69 and ATOM46.

All newly discovered ATOM complex subunits are predicted to contain a single transmembrane domain (Fig. 6a). Of all the subunits, ATOM14 is the only one that shows similarity to any of the Tom subunits, namely Tom22. However, this similarity is very limited, and whereas Tom22 has a large cytosolic and a short intermembrane space domain this is reversed in ATOM14. ATOM69 is superficially similar to Tom70. Both have the same molecular weight and multiple TPR-like motifs. However, ATOM69 in addition has an N-terminal CS/Hsp20-like domain and, in contrast to Tom70, which has an N-terminal membrane anchor, is tail-anchored. ATOM46 on the other hand has an N-terminal membrane anchor and contains an armadillo (ARM) repeat domain^28^. The predicted topology of ATOM69 and ATOM46 (Fig. 6a) was confirmed experimentally. Both proteins are recovered in the pellet when subjected to an alkaline carbonate extraction indicating that they are integral membrane proteins (Fig. 6b). Protease protection assays using isolated mitochondria show that ~80% of ATOM69 and ATOM46 but not of the intermembrane space protein Tim9, are accessible to added protease, indicating that a large domain of the proteins is exposed to the cytosol (Fig. 6c). Finally, we show that removal of the predicted transmembrane domains of ATOM69 and ATOM46 renders the two proteins soluble (Fig. 6d). In summary these experiments confirm the predicted topology of ATOM69 and ATOM46 as shown in Fig. 6a.

### ATOM69 and ATOM46 are novel protein import receptors

**Figure 7:**
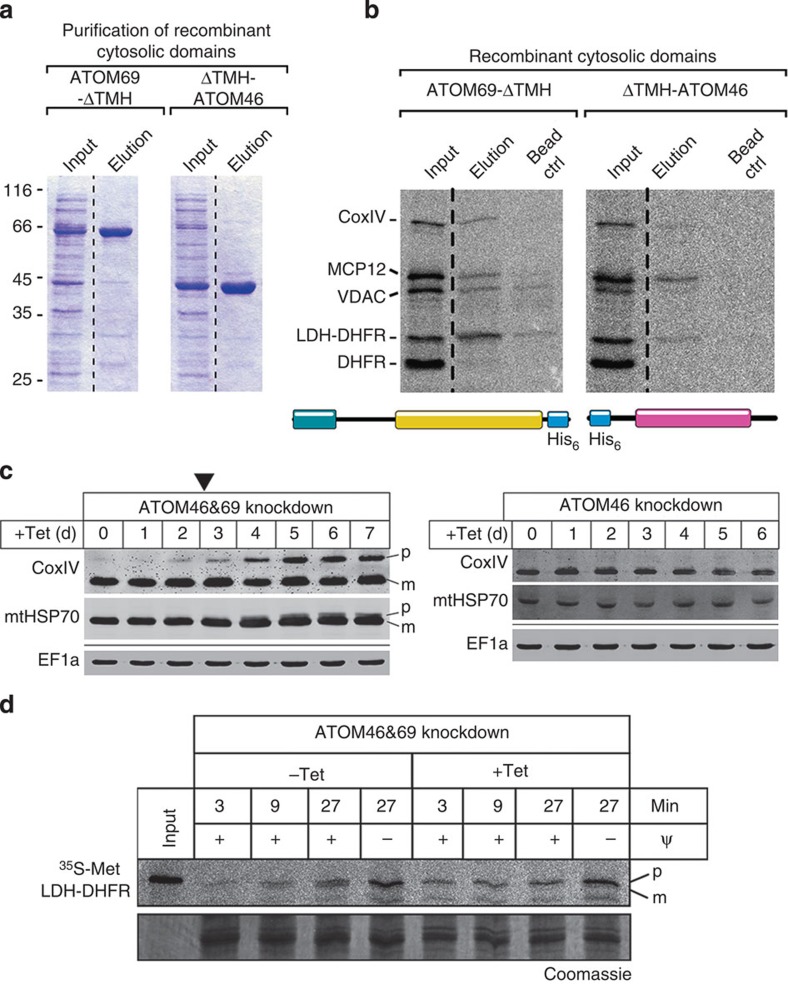
ATOM69 and ATOM46 are novel protein import receptors. (**a**) SDS–PAGE of purified His-tagged cytosolic domains of ATOM69 and ATOM46. (**b**) Binding of a mixture of precursor proteins to the cytosolic domains of ATOM69 and ATOM46. SDS–PAGE analysis showing 10% of the input fractions consisting of the indicated precursor proteins, the eluates from the Ni-NTA beads containing equimolar amounts of the indicated cytosolic domains and, as a control, the eluates from an equal volume of beads only. (**c**) Immunoblots of steady-state levels of CoxIV and mtHSP70 in whole-cell extracts of the ATOM69 and ATOM46-double knockdown cell line (left panel) or the ATOM46 knockdown cell line (right panel). Cytosolic EF1a serves as a loading control. Time of induction in days (d) is indicated at the top. The black triangle indicates the onset of the growth phenotype. The position of precursor (p) and mature forms (m) are indicated. (**d**) ^35^S-Met-labelled LDH-DHFR was imported into mitochondria isolated from the ATOM69 and ATOM46-double knockdown cell line. Ψ, membrane potential. Input, 10% of the added substrate, Coomassie-stained gels are shown as loading controls. The position of precursor (p) and mature forms (m) are indicated.

Their peripheral and in part exclusive association with the ATOM core complex (Fig. 5b) and the presence of protein–protein interaction domains in ATOM69 and ATOM46 (Fig. 6a) suggest they may function as mitochondrial protein import receptors. To test whether they can bind precursor proteins, the His-tagged cytosolic domains of the two proteins, termed ATOM69-ΔTMH and ΔTMH-ATOM46, were recombinantly expressed in *Escherichia coli* and purified (Fig. 7a). Subsequently a mixture of [^35^S]-labelled *in vitro* translated mitochondrial preproteins were incubated with the resin-bound cytosolic domains or as a control with resin only. After washing, bound proteins were eluted and analysed by SDS–PAGE and autoradiography (Fig. 7b). The results show that both cytosolic domains were able to bind mitochondrial preproteins. The binding was specific because DHFR was only bound when fused to the 14 amino acid long, presequence-containing amino-terminal part of LDH. Interestingly, ATOM69-ΔTMH and ΔTMH-ATOM46 show distinct but in part overlapping specificities. MCP12 was bound by both proteins with comparable efficiencies, whereas the two presequence-containing proteins pre-CoxIV and pre-LDH-DHFR and the voltage-dependent anion channel (VDAC) interacted preferentially with the ATOM69-ΔTMH.

In line with its broader substrate specificity, knockdown of ATOM69 causes a growth arrest and *in vivo* accumulation of precursor protein at late time points after knockdown induction (Fig. 3c). ATOM46 in contrast is dispensable for normal growth. However, *in vitro* import of LDH-DHFR, which can bind to both ATOM69-ΔTMH and ΔTMH-ATOM46, was not impaired in mitochondria isolated from the procyclic ATOM69 knockdown cell line.

Interestingly, in an ATOM69/ATOM46-double knockdown cell line these phenotypes are strongly exacerbated. In contrast to the ATOM69 knockdown cell line, accumulation of precursor proteins is observed much earlier (compare Figs 7c and 3c) and *in vitro* import of LDH-DHFR is abolished (Fig. 7d). This supports the notion that ATOM69 and ATOM46 are in part redundant mitochondrial protein import receptors with distinct but partially overlapping substrate specificity.

## Discussion

The general features of mitochondrial protein import machineries described in textbooks are essentially based on experiments that have been done in two fungal species only. To provide a more panoramic view on mitochondrial protein import, we have performed a comprehensive analysis of the ATOM complex of the protozoa *T. brucei*, which belongs to a different eukaryotic supergroup than the fungi. The ATOM complex is the first OM protein translocase that is characterized at this level outside the fungal clade and deviates to a surprising extent from the TOM translocase of all other eukaryotes. It consists of six subunits, five of which are essential in the two replicative stages of the parasites life cycle. Except for the pore-forming β-barrel protein ATOM40 (refs 14, 15, 18), which is discussed in the introduction, and ATOM14 which has some remote similarity to Tom22, none of its subunits are homologous to any TOM subunits of yeast or any other organism. In contrast orthologues for all six ATOM subunits are detected in all kinetoplastid species whose genomes have been sequenced (Supplementary Table 1).

Why is the trypanosomal ATOM complex so different from the classic TOM complex? Was it shaped by different functional constraints than the TOM complex? The estimated number of imported proteins in trypanosomes is ~1,000 und thus very similar to yeast^19^,^29^,^30^. Many of these protein carry presequences and even though the trypanosomal ones are generally short, they often can be predicted using the same algorithms that are used for other eukaryotes^31^, suggesting they have the same physicochemical features. In line with these findings, functional interchangeability of trypanosomal presequences with their counterparts in other eukaryotes has extensively been demonstrated both *in vivo* and *in vitro*^27^,^32^,^33^,^34^,^35^. Moreover, β-barrel proteins and mitochondrial carrier proteins, two groups of imported proteins that lack presequences, are also found in trypanosomes^16^,^19^,^36^. Interestingly, it has been shown that the trypanosomal orthologues of these proteins are correctly imported into yeast mitochondria and *vice versa*^35^,^37^.

Most mitochondria not only import proteins but also at least a few transfer RNAs (tRNAs)^38^. Trypanosomes are unique in this respect, they completely lack mitochondrial tRNA genes and have to import the whole set of organellar tRNAs from the cytosol^39^. However, whether import of tRNAs depends on the ATOM complex and whether this may have played a role in its evolution is presently unclear.

In summary, it is clear that functionally the trypanosomal ATOM complex is essentially equivalent to the TOM complex in yeast. It imports a large number of the same type of substrate proteins and likely recognizes the same targeting signals than the TOM complex. The structural differences between the ATOM and the TOM complex can therefore not be explained by functional differentiation but likely are due to independent evolutionary histories.

The divergent nature of the ATOM complex is important for two reasons. First, its five essential subunits may constitute attractive novel drug targets against kinetoplastid diseases, such as sleeping sickness and nagana caused by *T. brucei*^40^. Second and more significant in the context of our study, comparing the translocases of yeast and trypanosomes allows to define the basic features of a mitochondrial OM protein translocation machinery. Based on this comparison, we suggest that the prototype translocase consists of a core composed of a β-barrel protein (ATOM40 or Tom40) that forms the pore and an associated smaller protein with a single membrane-spanning domain (ATOM14 or Tom22), which stabilizes the pore and might regulate preprotein transfer. This core is associated with a number of small proteins (ATOM12/ATOM11 and Tom5/Tom6/Tom7) a conserved function of which is to regulate assembly or disassembly of the translocase subunits^41^. ATOM11 supports the association of the core complex with its receptor subunits ATOM69 and ATOM46. Tom6 has a somewhat similar function: it mediates binding of Tom40 with the secondary receptor Tom22. ATOM12 and Tom7 seem to have antagonistic functions to ATOM11 and Tom6, respectively. They facilitate the dissociation of the receptors ATOM69 and Tom20 from their respective core complexes.

The receptor subunits ATOM69 and ATOM46 were the main focus of our study. Their sequences, domain structures and, in the case of ATOM69, its topology indicate that they evolved independently from the classic protein import receptors Tom70 and Tom20. Thus, the two receptor pairs ATOM69/ATOM46 and Tom70/Tom20 represent different solutions to the same biological problem—namely to mediate mitochondrial import of a large number of different proteins—that have been implemented over very large phylogenetic distances.

ATOM69 is superficially similar to Tom70. Both have the same molecular weight and contain TPR-like motifs. Tom70 is a signal-anchored protein and has a large C-terminal cytosolic domain consisting of 11 TPR motifs. The eight TPR motifs distal to the membrane bind the substrate proteins, whereas the three TPR motifs proximal to the membrane form the clamp domain that mediates interaction with cytosolic Hsp70 or Hsp90 (refs 42, 43). Tom70 and ATOM69 have reciprocal topologies. ATOM69 has a large N-terminal cytosolic domain, which adjacent to the membrane anchor carries TPR-like motifs that may bind substrate proteins. ATOM69 has a CS/Hsp20-like domain close to the N terminus, which is distal to the membrane anchor. It has been shown for other proteins that this domain can bind to Hsp90 (ref. 44).

Thus, the mitochondrial OM translocase appears to require one receptor subunit that consists of three modules: a single membrane-spanning domain, a substrate binding domain consisting of multiple TPR motifs and a binding site for cytosolic chaperones that might or might not be based on TPR motifs. However, in which order the modules are arranged and whether the receptor is organized in a N_in_–C_out_ or N_out_–C_in_ orientation is not important provided that the protein-binding modules face the cytosol.

The presence of these overarching structural features that are shared between ATOM69 and Tom70 suggests that there are constraints imposed by functional selection of how a mitochondrial protein import receptors can be organized.

If ATOM69 is a functional analogue of Tom70 in yeast, ATOM46 might be an analogue of Tom20. Indeed, ATOM46 has a charged region near the C terminus that shows remote similarity to a short sequence of Tom20. However, the significance of this similarity is unclear because it is only detected when searching the PFAM data base using HMMER with relaxed search parameters. Moreover, unlike any other protein translocase subunits ATOM46 has an ARM repeat domain, which is a known protein–protein interaction module. ARM repeat domains are specific for eukaryotes and are found in a number of unrelated proteins, which include soluble nuclear transport receptors in which the ARM repeat domain binds the nuclear localization signals^28^.

While ATOM69, regarding some structural features, is more similar to Tom70 and ATOM46 to Tom20, the situation is different if we focus on functional aspects. In trypanosomes ATOM69 is essential and therefore more important than the dispensable ATOM46. ATOM69 in this respect resembles yeast Tom20 whose deletion causes a more severe phenotype than the lack of Tom70 (ref. 45). Furthermore, while the substrate specificities of the trypanosomal receptors need to be analysed in more detail, it is clear that ATOM69, similar to yeast Tom20, binds presequence-containing substrates more efficiently than ATOM46. Nevertheless, ATOM69 can bind the hydrophobic mitochondrial carrier protein MCP12. ATOM46, like Tom70, might be specialized for hydrophobic internal targeting sequences since the only substrate it could efficiently bind was MCP12.

**Figure 8:**
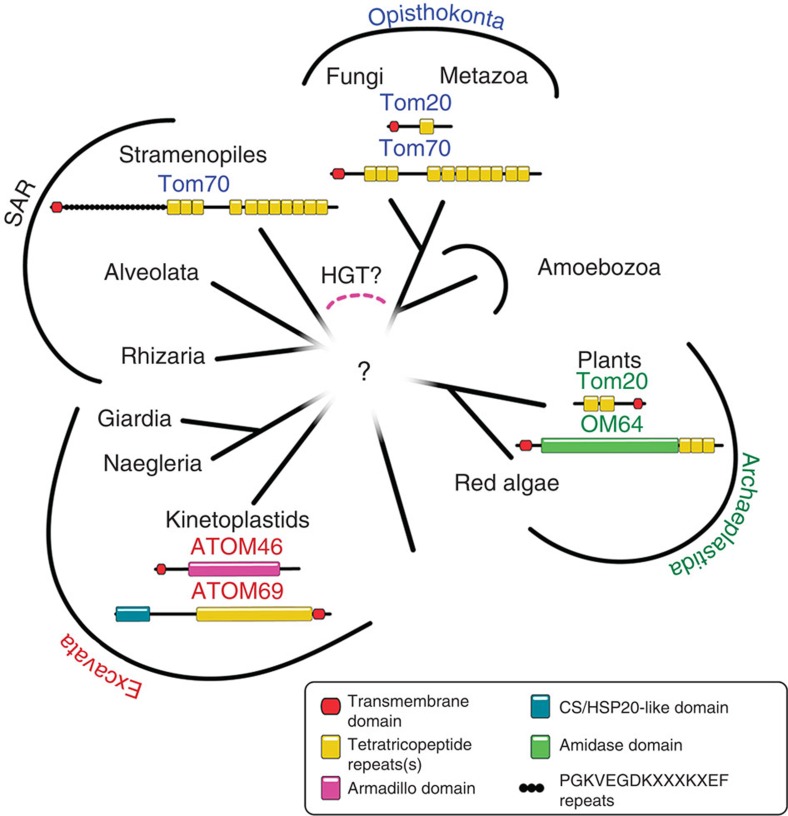
Diversity of mitochondrial outer membrane protein import receptors. Three pairs of receptors evolved independently in the Opisthokonta (Tom20/Tom70 shown in blue), in Archeaplastida (Tom20 and OM64 shown in green) and in Kinetoplastids (ATOM46/ATOM69 shown in red). Receptors are drawn to scale and protein domains are indicated. The Stramenopile Tom70 is an orthologue of the Tom70 found in the Opisthokonta, indicating that the Stramenopiles are related to the Opisthokonta or that the Stramenopile Tom70 was acquired by horizontal gene transfer (HGT). ATOM46 and ATOM69 have only been found in Kinetoplastids, indicating that other Excavata lost the receptors maybe due to reductive evolution or that the Excavata are not a monophyletic group.

The molecular characterization of the ATOM complex suggests a two step model for the evolution of the OM protein import system. First, a simple mitochondrial OM translocase consisting of a β-barrel protein of the mitochondrial porin family and a Tom22-like protein evolved in the endosymbiontic ancestor of the mitochondrion. At this time, the number of imported proteins was likely small and may not have required specialized import receptors. The fact that yeast lacking Tom20 and Tom70 are viable provided that the ‘secondary’ receptor Tom22 is present illustrates the plausibility of this scenario^45^. In a second step, specialized receptor subunits were recruited. Since we find different sets of receptors in different taxons, this must have happened after the segregation of the major eukaryotic supergroups (Fig. 8). The selective force that led to the evolution of specialized receptor pairs most likely was the need to import an ever larger number of mitochondrial proteins. This indicates that there was a potentially massive expansion of the mitochondrial importome that happened after the divergence of the eukaryotic supergroups. Finally, we end up with the present situation where >95% of all mitochondrial proteins are imported from the cytosol in a process that requires two functionally distinct receptors. We know of three such receptor pairs: (i) the classic import receptors Tom20 and Tom70, which are thought to be restricted to the Opisthokonts. (ii) OM64 and Tom20, which function as receptors in plant mitochondria^46^. OM64 is a large mitochondrial OM protein, which contains an amidase-like domain and multiple TPR motifs^46^. Plant Tom20 binds presequences and is structurally and functionally similar to yeast Tom20 but coded in reverse^12^. Instead of having a N_in_–C_out_ plant, Tom20 has a N_out_–C_in_ orientation indicating that it evolved independently of yeast Tom20. (iii) ATOM69 and ATOM46 the two import receptors of Kinetoplastids, which belong to the supergroup of the Excavates.

The finding of three distinct receptor pairs that evolved independently from each other and that are restricted to single supergroups, each strongly support that import receptors are a late addition to the OM translocase that evolved independently (Fig. 8). However, there are some caveats. Recently, it was suggested that the mitochondria-related organelle of some Stramenopiles may have a remote Tom70 orthologue^11^. This suggests that the Stramenopiles, which belong to the newly coined supergroup SAR (consisting of the Stramenopiles, Alveolata and Rhizaria) and the Opisthokonts, are closely related to each other to the exclusion of the other supergroups. However, there is also the possibility that this unexpected result could be explained by horizontal gene transfer.

In summary, our study shows that a comparative structural and functional analysis of mitochondrial OM translocases in the different eukaryotic supergroups provides insight into the evolution of the mitochondrial protein import system, a key event in the conversion of the endosymbiont into an organelle. Our study also illustrates that tracking down the evolutionary history of mitochondrial OM import receptors is a suitable approach to reveal the deep-branching relationships of basic eukaryotic taxa that ultimately will help to shed light on the origin of the eukaryotic cell.

## Methods

### Transgenic cell lines

Transgenic procyclic cell lines are based on *T. brucei* 427 or 29-13 (ref. 47) and were grown at 27 °C (or where indicated at 33 °C) in SDM-79 supplemented with 5 or 10% (vol/vol) foetal calf serum (FCS), respectively. Transgenic bloodstream form cell lines are based on the New York single marker strain^47^ or a derivative thereof termed F_1_γL262P (ref. 26). Bloodstream form cells were cultured at 37 °C in HMI-9 containing 10% FCS.

One ATOM40 allele was tagged *in situ* at the C terminus with a triple HA-epitope^48^, either in *T. brucei* 427 for IP experiments analysed by mass spectrometry (Fig. 1a) or in *T. brucei* 29-13 expressing full length c-Myc-tagged ATOM complex subunit candidate proteins for digitonin extractions and immunofluorescence (Fig. 1b,c) or reciprocal IP analysis (Supplementary Fig. 1). For inducible triple c-Myc-tagging of candidate proteins, the full length open reading frames (ORFs) of ATOM11, ATOM12, ATOM14, ATOM46 and ATOM69 or truncated versions of ATOM69 (ORF nt 1-1695) and ATOM46 (ORF nt 109-1260) lacking the transmembrane domain were cloned into modified pLew100 (ref. 47) expression vectors in which the phleomycine resistance gene had been replaced by a puromycine resistance gene and triple c-Myc cassettes^48^ had been inserted to allow for N- or C-terminal positioning of the tag using BamHI, HindIII or AgeI restriction sites.

RNA interference (RNAi) was done using pLew100-derived stemloop vectors^47^,^49^ in which the phleomycine resistance gene had been replaced by a blasticidine resistance gene and which allow for ligation of inserts in opposing directions separated by a 460 bp spacer fragment using BamHI/XhoI and HindIII/XbaI restriction sites^49^. The following inserts were used: ATOM12 (ORF nt 13-314), ATOM14 (full ORF), ATOM46 (ORF nt 226-664) and ATOM69 (ORF nt 687-1113). ATOM11 in bloodstream form cells was downregulated using an RNAi construct targeting the 3′ untranslated region (nt 22-612 after the stop codon). In procyclic forms, a conditional knockout cell line for ATOM11 was constructed by introduction of an ectopic inducible C-terminally triple c-Myc-tagged copy of the gene and sequential replacement of both alleles by phleomycine and blasticidine resistance genes. Successful knockout of both wild-type ATOM11 alleles has been confirmed by PCR (Supplementary Fig. 5). A list of primers is provided (Supplementary Table 2).

### IPs and mass spectrometry

Isotonically isolated mitochondria (600 μg)^27^ from wild-type cells or cells expressing C-terminally HA-tagged ATOM40 were solubilized for 15 min on ice in 600 μl lysis buffer: 20 mM Tris-HCl pH 7.4, 0.1 mM EDTA, 50 mM NaCl, 10% glycerol, 1 mM phenylmethylsulfonylfluoride (PMSF), 1.5% (w/v) digitonin, complete protease inhibitor cocktail, EDTA-free (Roche Applied Science). The lysate was cleared by centrifugation (18,000*g*, 4 °C) and the supernatant was incubated for 2 h at 4 °C with 100 μl of a 1:1 slurry of anti-HA agarose (Roche Applied Science, Product No. 11815016001). Beads were washed 10 times in 500 μl lysis buffer containing 0.5% (w/v) digitonin before elution for 5 min at 95 °C with 75 μl of 60 mM Tris-HCl pH 6.8, 0.1% SDS. For mass spectrometric analysis, the samples were precipitated using acetone and tryptically digested in 60% (v/v) methanol and 20 mM NH_4_HCO_3_. The resulting peptides were analysed by nano-HPLC/ESI-MS/MS as described^50^. For elution under native conditions, beads were incubated for 15 min at 25 °C with 20 mM Tris-HCl pH 7.4, 0.1 mM EDTA, 100 mM NaCl, 25 mM KCl, 10% glycerol, 0.2% (w/v) digitonin containing 1 mg ml^−1^ HA peptide. Eluates of ATOM40-HA and control IPs were subjected to BN–PAGE on a 4–13% gel. The gel was silver stained, lanes (molecular weight range 440 kDa and higher) cut into equal slices and destained using 60 mM K_3_[Fe(CN)_6_]/25 mM Na_2_S_2_O_3_. Proteins were digested in-gel with trypsin (37 °C, overnight) and analysed by liquid chromatography mass spectrometry as described before^51^ using an UltiMate 3000 RSLCnano system (Dionex LC Packings/Thermo Fisher Scientific, Idstein, Germany) coupled to an LTQ-Orbitrap XL mass spectrometer (Thermo Fisher Scientific, Bremen, Germany). Peptide mixtures were separated on a 15-cm C18 reversed-phase nano LC column applying a linear 30-min gradient ranging from 4 to 34% (v/v) acetonitrile in 0.1% (v/v) formic acid. The column temperature was 60 °C and the flow rate was 300 nl min^−1^. MS survey scans were acquired in the orbitrap (*m/z* 370—1,700; resolution of 60,000 at *m/z* 400). Simultaneously, the five most intense peptide ions with a charge of ≥+2 were subjected to fragmentation by low-energy collision-induced dissociation in the linear ion trap applying a normalized collision energy of 35% with an activation *q* of 0.25 and an activation time of 30 ms. The dynamic exclusion time for previously selected precursor ions was 45 s. The instrument was externally calibrated with standard compounds on a routine basis to ensure for accurate mass measurements (mass error ≤2 p.p.m.). For peptide and protein identification, MS data sets were correlated with the TriTryp database 3.1 using MaxQuant, version 1.0.13.13 (ref. 52), in combination with Mascot (version 2.2, Matrix Science)^53^ as a search engine. A false discovery rate of <1% was applied, and the MS intensity determined by MaxQuant for each protein was used as a quantitative measure.

For the verification of interactions between ATOM40-HA and the c-Myc-tagged candidate proteins, 1 × 10^8^ Tet-induced (1 day) cells were incubated for 5 min on ice in 20 mM Tris-HCl pH 7.5, 0.6 M sorbitol, 2 mM EDTA containing 0.015% (w/v) digitonin. After centrifugation (6,800*g*, 4 °C) ,the resulting mitochondria-enriched pellet was solubilized on ice for 15 min in 200 μl lysis buffer: 20 mM Tris-HCl pH 7.4, 0.1 mM EDTA, 100 mM NaCl, 25 mM KCl and 1.5% (w/v) digitonin, containing the same protease inhibitors mentioned above. The lysate was cleared by centrifugation (20,800*g*, 4 °C) and the supernatant was incubated for 2 h at 4 °C in presence of 40 μl of 1:1 slurry anti-c-Myc agarose (Clontech Laboratories, Inc., Product No. 631208) or 50 μl of 1:1 slurry of anti-HA agarose. Beads were washed three times with 500 μl lysis buffer containing 0.2% (w/v) digitonin prior to elution by boiling in SDS-gel loading buffer.

### *In vitro* protein import

^35^S-Met-labelled LDH–DHFR was synthesized using the TNT T7 Quick for PCR (Promega) *in vitro* translation kit according to the instruction manual. For the coupled transcription and translation, a plasmid encoding the N-terminal 150 amino acids of LDH fused to mouse DHFR under the control of a T7 promoter was used as a template^14^. Isotonically isolated mitochondria from uninduced and induced knockdown cells, harvested prior to the appearance of the growth phenotype, were prepared and *in vitro* protein import was essentially done as described^27^. Briefly, 25μg mitochondria were taken up in import buffer (20 mM HEPES-KOH, pH 7.4, 0.6 M sorbitol, 25 mM KCl, 10 mM MgCl_2_, 1 mM EDTA, 2 mM KH_2_PO_4_, 5 mg ml^−1^ fatty acid-free BSA) containing 4 mM ATP, 0.5μg creatine kinase and 20 mM phosphocreatine. ^35^S-Met-labelled LDH-DHFR was added to the reaction for the indicated times. The total reaction volumes were 25μl. The membrane potential was disrupted and import reactions were stopped by the addition of 4μM valinomycin and 100 μM carbonyl cyanide 3-chlorophenylhydrazone. All samples were treated with proteinase K at an end concentration of 80μg ml^−1^ followed by the addition of 4 mM PMSF and reisolation of mitochondria by centrifugation. Full scans of autoradiographs and gels are shown in Supplementary Fig. 6.

### Antibodies

Full length His_6_- or MBP-tagged ATOM11, ATOM14, ATOM46 and ATOM69 were recombinantly expressed in *E. coli*. Proteins were either isolated by affinity chromatography or purification of inclusion bodies. Purified proteins were separated on SDS–PAGE and the Coomassie-stained bands were cut out and used to produce polyclonal rabbit antisera commercially (Eurogentec, Belgium). The ATOM14 serum was used at a dilution of 1:500. ATOM11, ATOM46 and ATOM69 sera were subjected to affinity purification using the recombinant proteins. Purified antibodies were used at a dilution of 1:50. Other antibodies used in this study were: mouse anti-c-Myc (Invitrogen, Product No. 132500, dilution 1:2,000), rabbit anti-c-Myc (Bethyl Laboratories, Inc., Product No. A190-105 A, dilution 1:1,000), mouse anti-HA (Enzo Life Sciences AG, Product No. CO-MMS-101 R-1000, dilution 1:5,000) and mouse anti-EF1a (Merck Millipore, Product No. 05-235, dilution 1:10,000). Polyclonal rabbit anti-VDAC (dilution 1:1,000), anti-ATOM40 (dilution 1:1,000), anti-CoxIV (dilution 1:1,000) and anti Cyt *c*1(dilution 1:1,000) were previously produced in our lab^19^. Mouse anti-REAP1 (ref. 54; dilution 1:1,500), rabbit anti-mtHSP70 (ref. 55; 1:1,000) and rabbit anti-MCP5 (ref. 37; dilution 1:2,500) were kindly provided by S. H. Hajduk, R. Jensen and F. Voncken, respectively. Full scans of blots are shown in Supplementary Fig. 6.

### Protease protection assay

Isotonically isolated mitochondria (25 μg each) were resuspended in 20 mM Tris-HCl pH 7.2, 15 mM KH_2_PO_4_, 20 mM MgSO_4_, 0.6 M sorbitol in a total volume of 50 μl with the indicated additions of proteinase K (10 mg ml^−1^) and 0.5% (v/v) Triton-X100 followed by incubation on ice for 15 min. Reactions were stopped by adding PMSF at 5 mM and mitochondria were centrifuged (6,800*g*, 4 °C), resuspended in SDS loading buffer and boiled. Full scans of blots are shown in Supplementary Fig. 6.

### Carbonate extractions

Isotonically isolated mitochondria (100 μg each) were resuspended in 160 μl of 100 mM Na_2_CO_3_ pH 11.5. About 80 μl were removed and mixed with 40 μl 3 × SDS loading buffer and boiled to serve as the ‘total’ sample. The remaining 80 μl was incubated on ice for 10 min and centrifuged (100,000*g*, 4 °C, 10 min.). The pellet was resuspended in 80 μl of 100 mM Na_2_CO_3_. All samples were analysed by SDS–PAGE. Full scans of blots are shown in Supplementary Fig. 6.

### Binding of precursors to cytosolic receptor domains

The cytosolic domains of ATOM69 (amino acids: 1–569) and of ATOM46 (amino acids: 37–419) were fused to a C-terminal and N-terminal hexahistidine tag, respectively, expressed in *E. coli* and purified by Ni-affinity chromatography. The purity of the isolated protein was assessed by SDS–PAGE (Fig. 7a).

The radioactive precursor proteins, pre-LDH (1-14)-DHFR, VDAC (Tb927.2.2510), pre-CoxIV (Tb927.1.4100) and MCP5 (Tb927.10.14810) were synthesized as described for ‘*in vitro* protein import’ and mixed in a ratio yielding equal radioactive intensities.

Binding assays were essentially done as described^9^. In short, for each reaction a bead volume containing 0.5 nmol of the bound proteins or the same volume of control beads was used. The beads were washed three times with 450 μl of binding buffer (20 mM imidazole, 100 mM KCl, 10 mM MOPS-KOH pH 7.2, 1% (w/v) BSA and 0.5% (w/v) digitonin) and then resuspended in 93 μl of binding buffer. To each reaction, 7 μl of precursor protein mix was added and incubated at 27 °C for 40 min. The beads were washed three times with 450 μl of 20 mM imidazole, 100 mM KCl, 10 mM MOPS-KOH pH 7.2, 0.1% (w/v) digitonin and eluted with two times 100 μl of elution buffer (50 mM NaH_2_PO_4_, 300 mM NaCl, 500 mM imidazole pH 8) The eluted proteins were trichloroacetic acid (TCA) precipitated and analysed by 14% SDS–PAGE. Full scans of autoradiographs and gels are shown in Supplementary Fig. 6.

### Immunofluorescence microscopy

Expression of triple c-Myc-tagged ATOM14, ATOM69, ATOM11, ATOM12 and ATOM46 was induced for 24 h. Cells were fixed with 4% paraformaldehyde in PBS and permeabilized with 0.2% Triton-X100 in PBS. Primary antibodies were mouse anti-c-Myc and rabbit anti-ATOM40 (1:1,000) and secondary antibodies were goat anti-mouse IRDye680RD conjugated (LI-COR Biosciences, Product No. 926-68070, dilution 1:500) and goat anti-rabbit FITC conjugated (Sigma, Product No. F0382, dilution 1:100). Cells were postfixed in cold methanol and slides mounted with VectaShield containing 4',6.diamidino-2-phenylindole (DAPI) (Vector Laboratories, Product No. H-1200). Images were acquired with a DFC360 FX monochrome camera (Leica Microsystrems) mounted on a DMI6000B microscope (Leica Microsystems). Images were analysed using LAS AF software (Leica Microsystems).

### Digitonin extractions

Plasmamembranes were lysed by resuspension of cells in SoTe buffer (20 mM Tris-HCl pH 7.5, 0.6 M sorbitol and 2 mM EDTA) containing 0.015% (w/v) digitonin followed by differential centrifugation. This yielded a mitochondria-enriched pellet fraction and a fraction enriched for cytosolic proteins^49^. Full scans of blots are shown in Supplementary Fig. 6.

### Northern blotting

Total RNA was isolated using acid guanidinium thiocyanate–phenol–chloroform extraction^56^. RNA was separated on a 1% agarose gel in 20 mM MOPS buffer, pH7.0 containing 0.5% formaldehyde. Northern probes were prepared from gel-purified PCR products corresponding to RNAi inserts described above and radioactively labelled using the Prime-a-Gene labelling system (Promega). Full scans of blots and gels are shown in Supplementary Fig. 6.

### BN–PAGE

Mitochondrial membranes were solubilized in a buffer (20 mM Tris-HCl pH 7.4, 50 mM NaCl, 10% glycerol and 0.1 mM EDTA) containing 1.5% (w/v) digitonin. Solubilized membrane extracts were cleared by centrifugation prior to separation on 4–13% gradient gels. To facilitate transfer of proteins to membranes, gels were incubated in SDS–PAGE running buffer (25 mM Tris, 1 mM EDTA, 190 mM glycine, 0,05% (w/v) SDS) prior to Western blotting. Full scans of blots are shown in Supplementary Fig. 6.

## Author contributions

J.M., S.D., M.N., O.S., M.P. and C.G. designed, performed and analysed all experiments except for mass spectrometric analyses, which were done by S.O.; A.C. provided technical support; A.S., B.W. and C.M. supervised the project; A.S. coordinated the entire project and obtained the main source of funding; J.M. prepared the figures; A.S. and J.M. wrote and revised the manuscript.

## Additional information

**How to cite this article:** Mani J. *et al.* Mitochondrial protein import receptors in Kinetoplastids reveal convergent evolution over large phylogenetic distances. *Nat. Commun.* 6:6646 doi: 10.1038/ncomms7646 (2015).

## Supplementary Material

Supplementary Information Supplementary Figures 1-6, Supplementary Tables 1-2 and Supplementary References

Supplementary Data 1 Proteins identified in ATOM40-HA complexes immunoprecipitated under denaturing conditions. Proteins identified with at least three evidence counts in both replicates in the ATOM40-HA pulldown only (i.e. absent from the corresponding wild type control; “ATOM40-HA only”) or with an MS intensity ratio ATOM40-HA/wild type of > 3 and exhibiting a sequence coverage of ≥ 5% and a posterior error probability (PEP) of < 0.01 were defined as candidate proteins. n.i., not identified.

Supplementary Data 2 Proteins identified in ATOM40-HA complexes immunoprecipitated under native conditions. Proteins identified with at least three evidence counts in the ATOM40-HA pulldown only (i.e. absent from the wild type control; “ATOM40-HA only”) or with an MS intensity ratio ATOM40-HA/wild type of > 5 and exhibiting a sequence coverage of ≥ 5% and a posterior error probability (PEP) of < 0.01 were defined as candidate proteins.
