# Supplementary material for: Mitochondrial protein import receptors in Kinetoplastids reveal convergent evolution over large phylogenetic distances
Source: Nat Commun. 2015 Mar 26;6:6646. doi: 10.1038/ncomms7646 (PMC4389251; doi:10.1038/ncomms7646)
Supplement: Supplementary Information — Supplementary Figures 1-6, Supplementary Tables 1-2 and Supplementary References [file ncomms7646-s1.pdf]

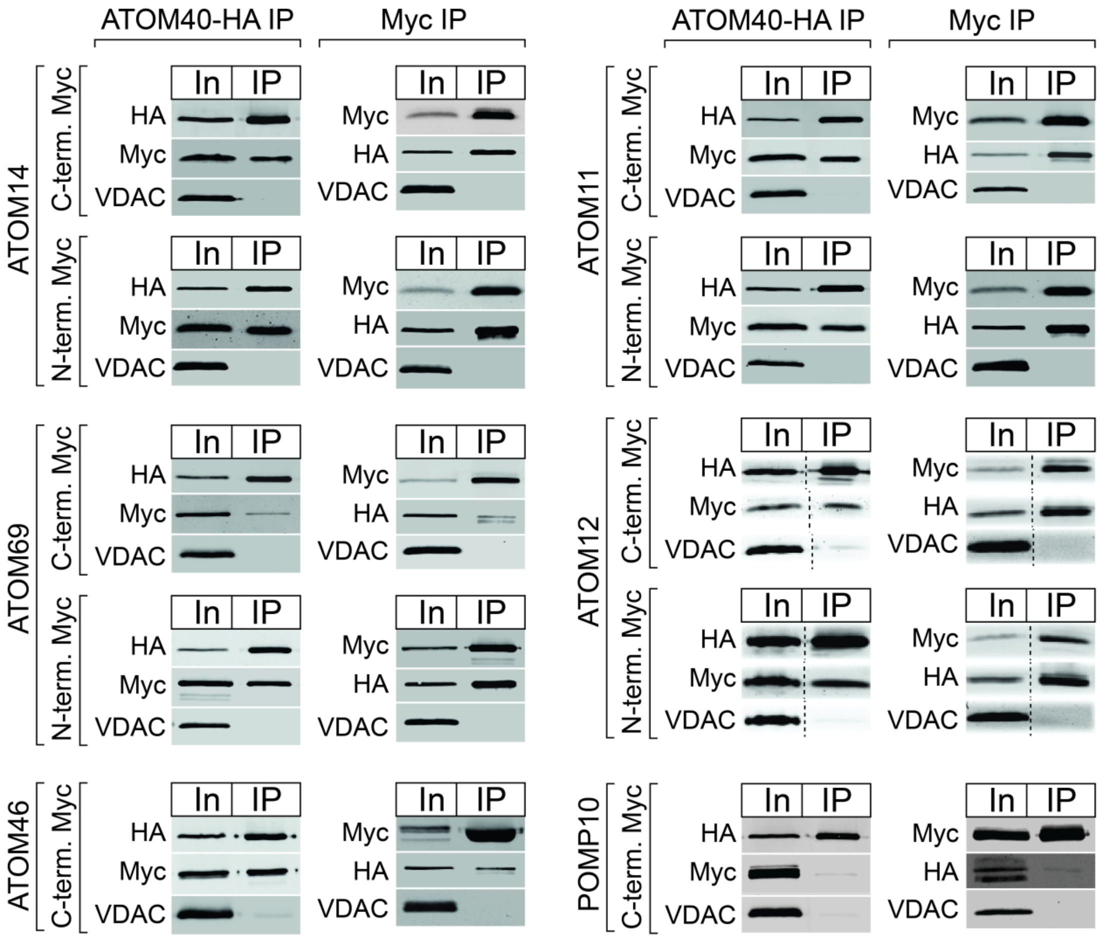

Supplementary Figure 1. Reciprocal IPs of ATOM40 and ATOM complex candidate subunits.

Mitochondrial membrane extracts of cell lines co-expressing C-terminally HA-tagged ATOM40 with the indicated N- or C-terminally c-Myc tagged ATOM complex candidate subunits were subjected to IPs using anti HA or anti c-Myc antisera, respectively. Immunoblots containing 5% of the input (In) and 100% of the eluate (IP) were probed for the presence of HA-tagged ATOM40 and the indicated c-Myc-tagged ATOM complex subunits. The abundant mitochondrial OM protein VDAC served as a control. POMP10 (Tb11.01.4740) which was one of 12 proteins present in the intersection between the OM proteome and the IP with subsequent denaturing elution (Fig. 1a) was used as a negative control.

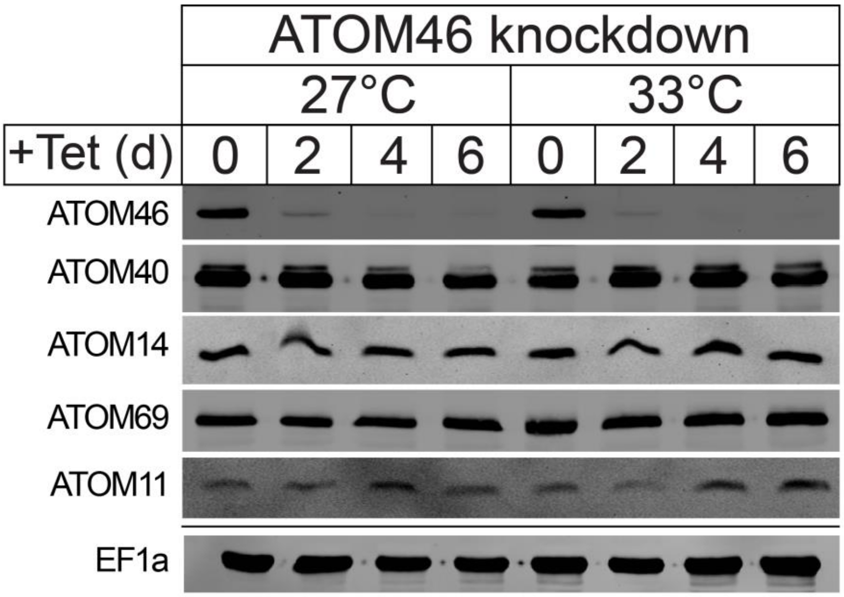

Supplementary Figure 2. Levels of ATOM complex subunits in ATOM46 knockdown cell line

Immunoblots showing the steady-state levels of ATOM complex subunits in whole cell extracts of the ATOM46 knockdown cell line grown at 27°C and 33°C, respectively. Cytosolic EF1a serves as a control. Time of induction in days (d) is indicated at the top.

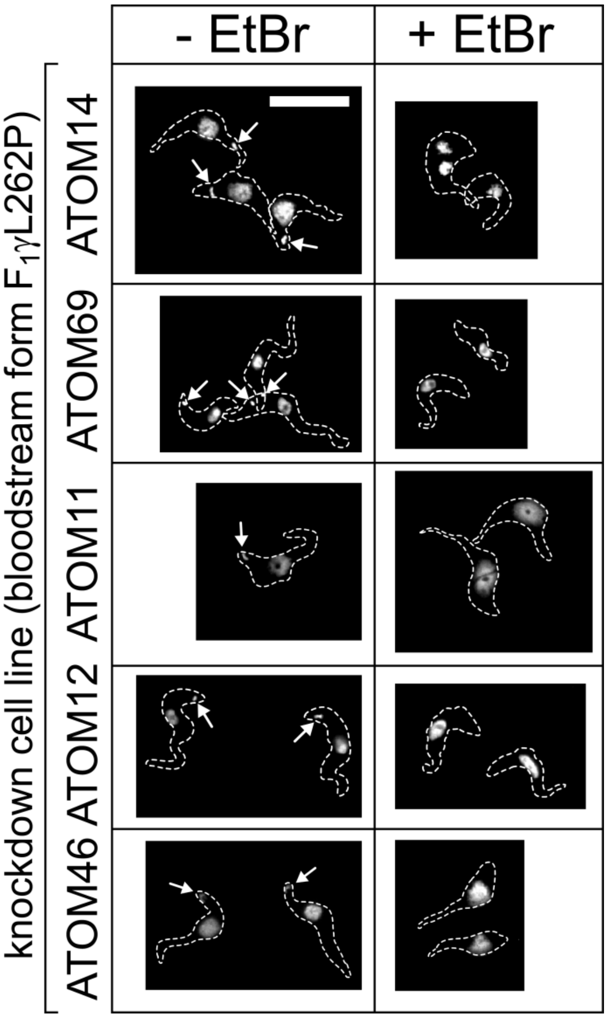

Supplementary Figure 3. Confirmation of the loss of kDNA in the ethidium bromide-treated $F_1\gamma$L262P knockdown cell lines. Uninduced $F_1\gamma$L262P knockdown cell lines for the indicated ATOM complex subunits untreated (-EtBr) and treated with ethidiumbromide (+EtBr) were stained with DAPI to visualize the nucleus and the kDNA. Arrows indicate the kDNA. The outlines of the cells have been traced in the phase contrast channel and are projected on the fluorescent images. Top left panel, scale bar corresponds to 12μm. All fluorescent images were equally scaled.

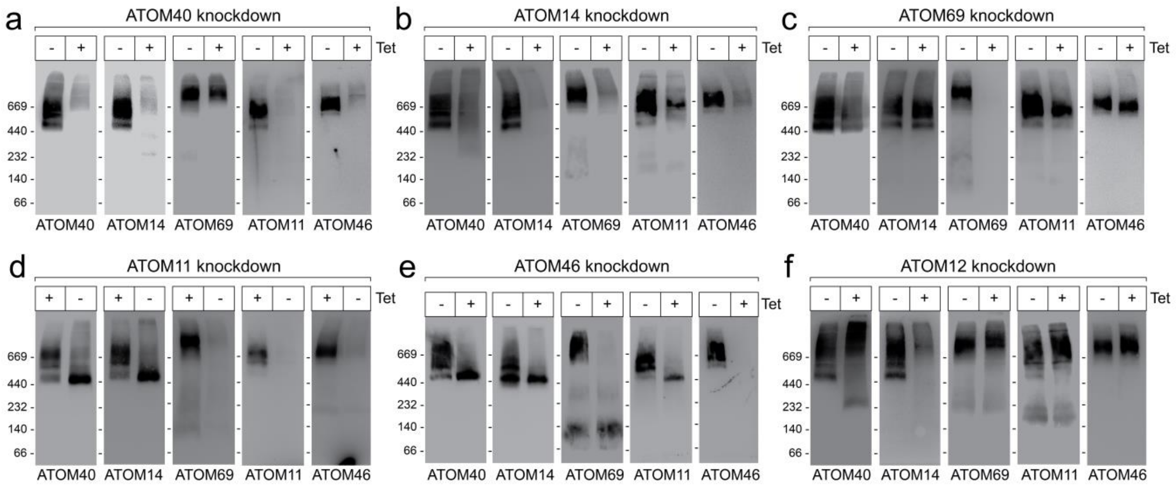

Supplementary Figure 4. Structural and functional interactions between ATOM complex subunits. (**a** to **f**) BN-PAGE immunoblots of mitochondrial membrane extracts of the indicated uninduced (-Tet) and induced (+Tet) ATOM complex subunit knockdown cell lines were probed with antisera against ATOM40, ATOM14, ATOM69, ATOM11 and ATOM46, respectively.

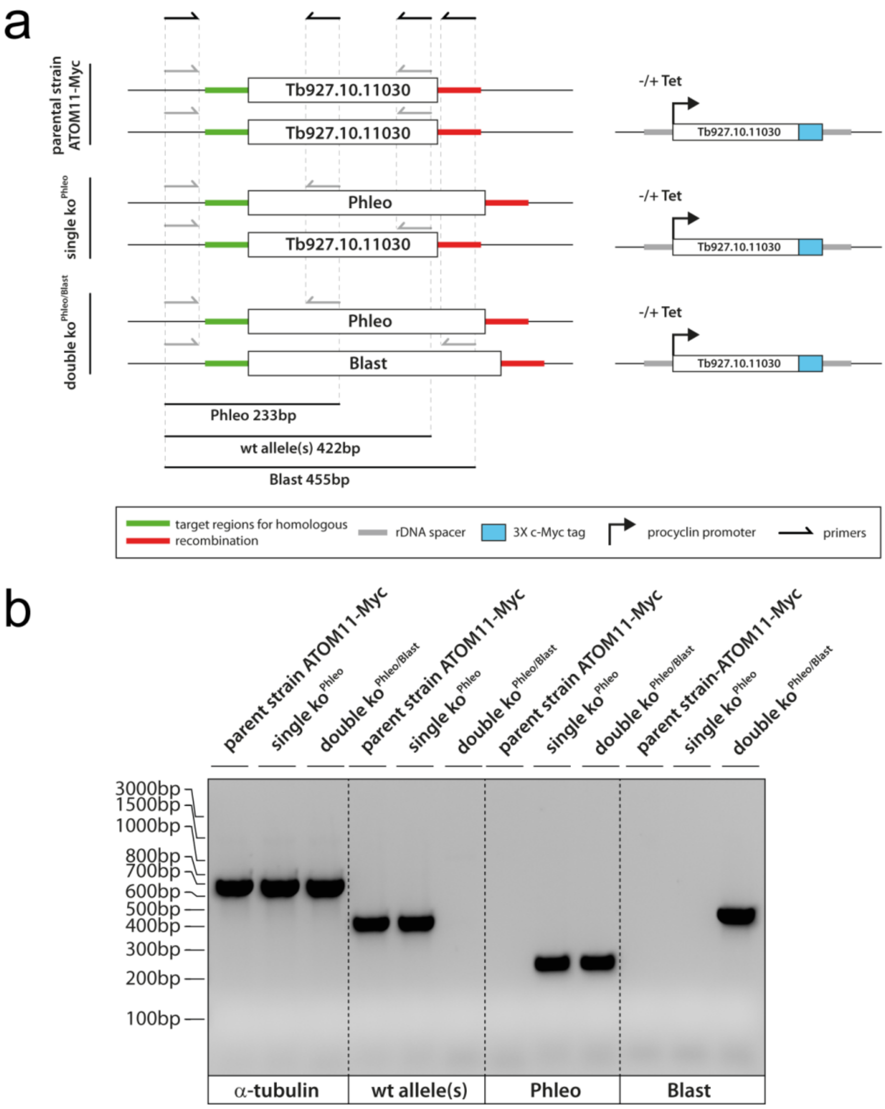

Supplementary Figure 5. Verification of successful replacement of both wildtype alleles of ATOM11 in the ATOM11 conditional knockout cell line. (**a**) Schematic drawing of the ATOM11 loci on chromosome 10 in the parental strain and after sequential replacement of the wildtype alleles with the phleomycine (Phleo) and blasticidine (Blast) resistance genes by homologous recombination. An inducible ectopic triple c-Myc tagged copy of ATOM11 had been introduced into the parental cell line prior to the knockouts. Positions of the loci-specific sense and ATOM11-, Phleo- and Blast-specific antisense primers, that were used to verify the integration events, are indicated. The sizes of the expected PCR products are indicated at the bottom. (**b**) Ethidium bromide-stained agarose gel of a PCR analysis of genomic DNA from the parental strain (parental strain ATOM11-Myc), single (single ko^{Phleo}) and double (double ko$^{Phleo/Blast}$) knockout cells. Left panel, amplification of a fragment of the α-tubulin gene showing that equal amounts of genomic DNA were analysed for all cell lines.

Figure 1c:

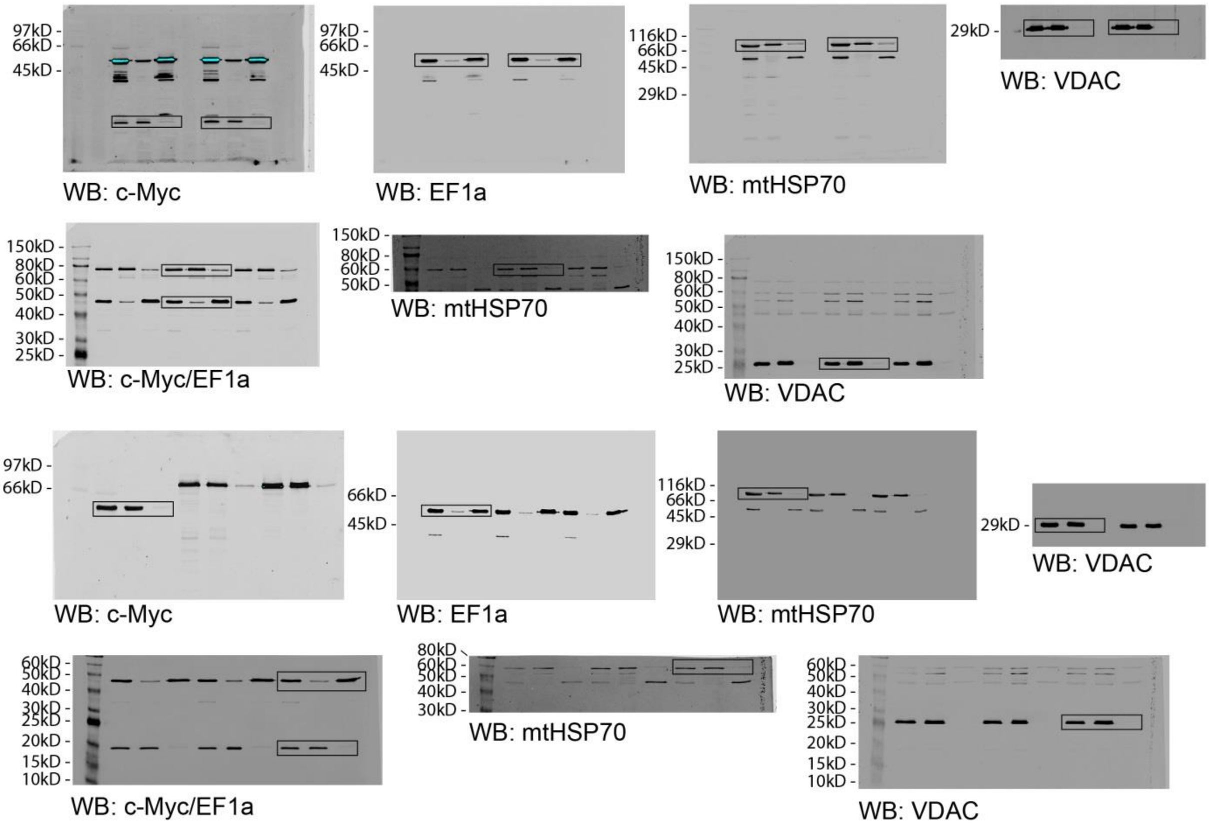
97kD
66kD
45kD
WB: c-Myc
97kD
66kD
45kD
WB: EF1a
116kD
66kD
45kD
29kD
WB: mtHSP70
29kD
WB: VDAC
150kD
80kD
60kD
50kD
40kD
30kD
25kD
WB: c-Myc/EF1a
150kD
80kD
60kD
50kD
WB: mtHSP70
150kD
80kD
60kD
50kD
40kD
30kD
25kD
WB: VDAC
97kD
66kD
WB: c-Myc
66kD
45kD
WB: EF1a
116kD
66kD
45kD
29kD
WB: mtHSP70
29kD
WB: VDAC
60kD
50kD
40kD
30kD
25kD
20kD
15kD
10kD
WB: c-Myc/EF1a
80kD
60kD
50kD
40kD
30kD
WB: mtHSP70
60kD
50kD
40kD
30kD
25kD
20kD
15kD
10kD
WB: VDAC

Figure 2:

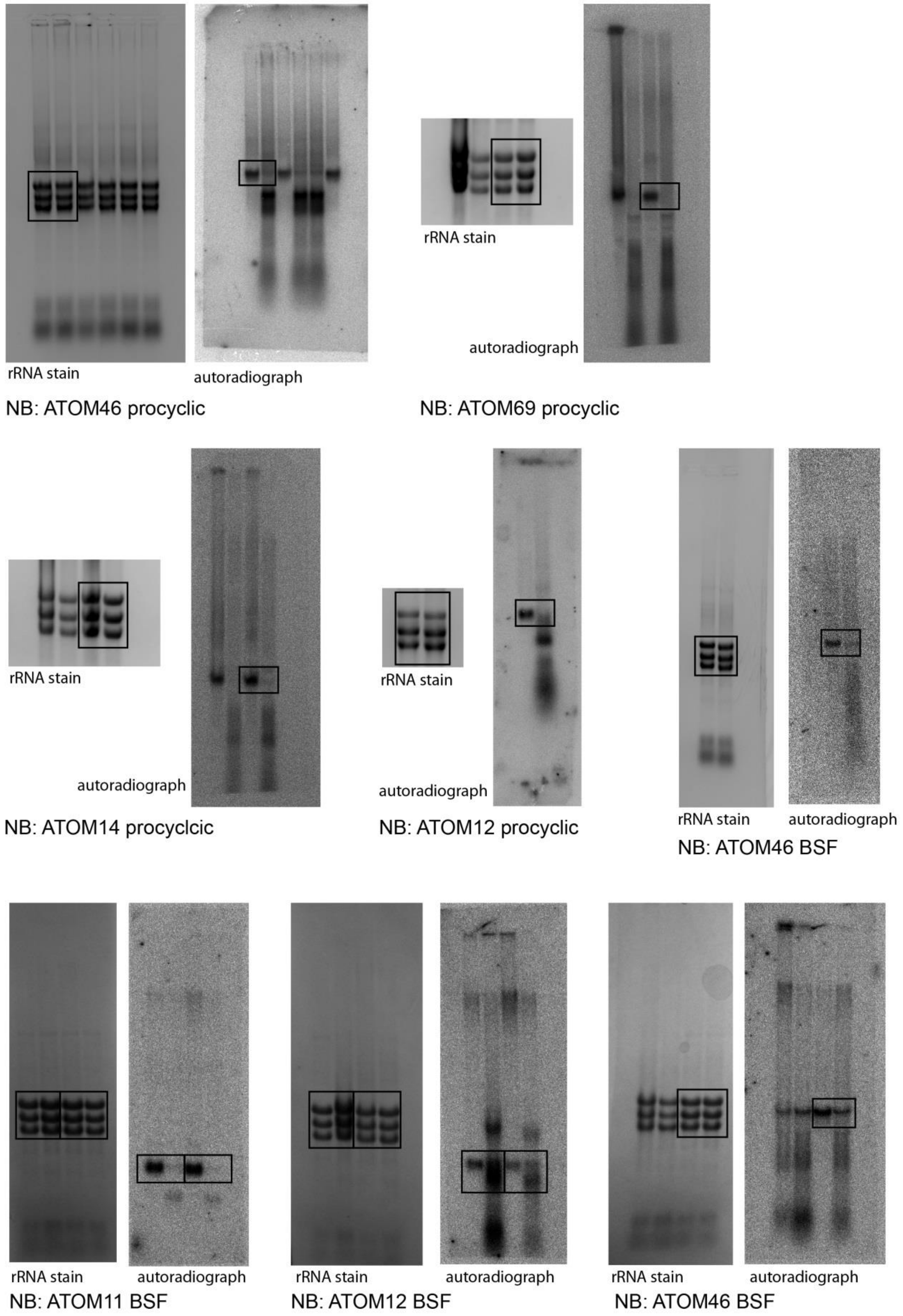
rRNA stain
autoradiograph
NB: ATOM46 procyclic
rRNA stain
autoradiograph
NB: ATOM69 procyclic
rRNA stain
autoradiograph
NB: ATOM14 procyclcic
rRNA stain
autoradiograph
NB: ATOM12 procyclic
rRNA stain
autoradiograph
NB: ATOM46 BSF
rRNA stain
autoradiograph
NB: ATOM11 BSF
rRNA stain
autoradiograph
NB: ATOM12 BSF
rRNA stain
autoradiograph
NB: ATOM46 BSF

Figure 2 cont.:

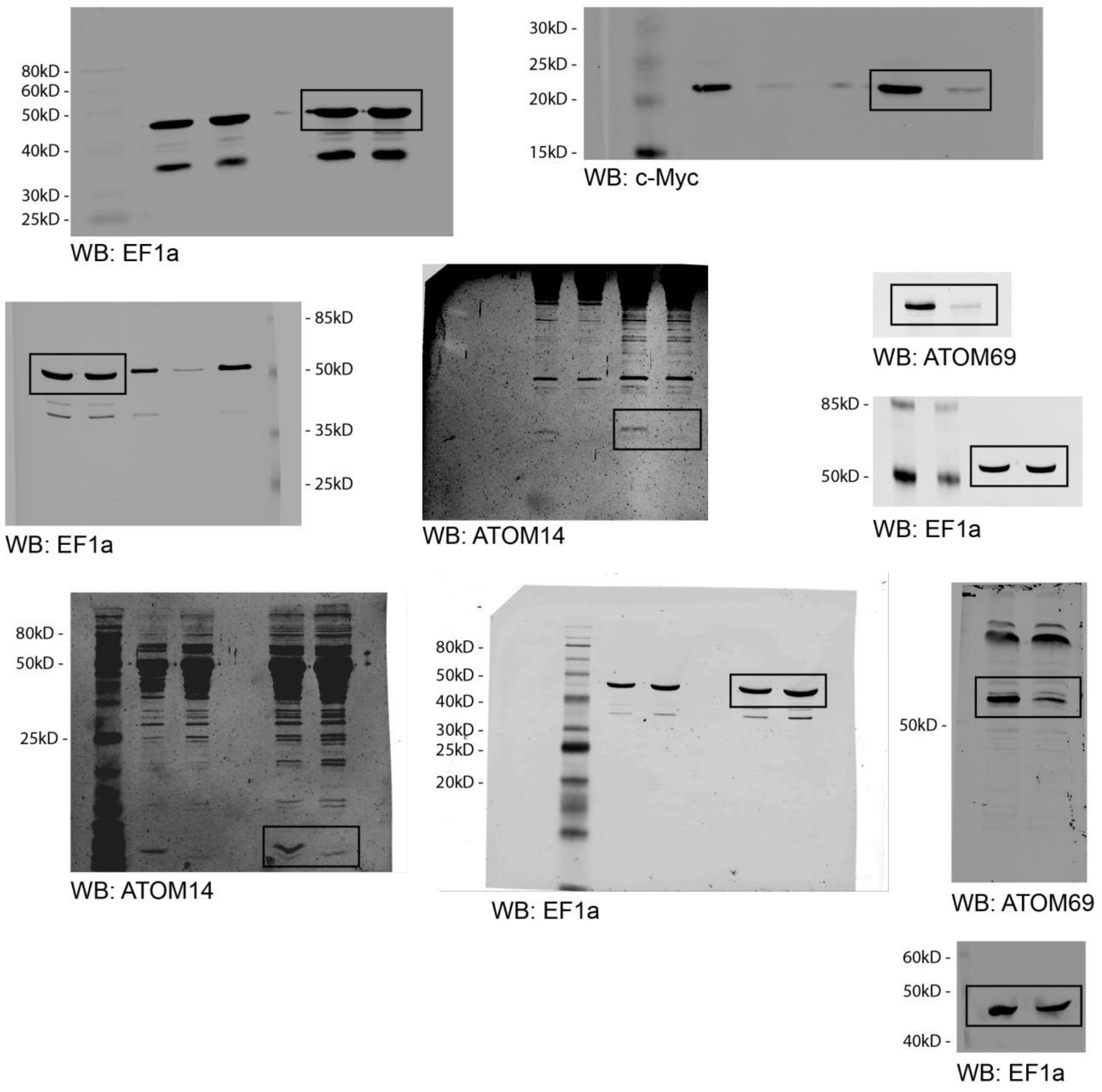

Figure 3a left panel:

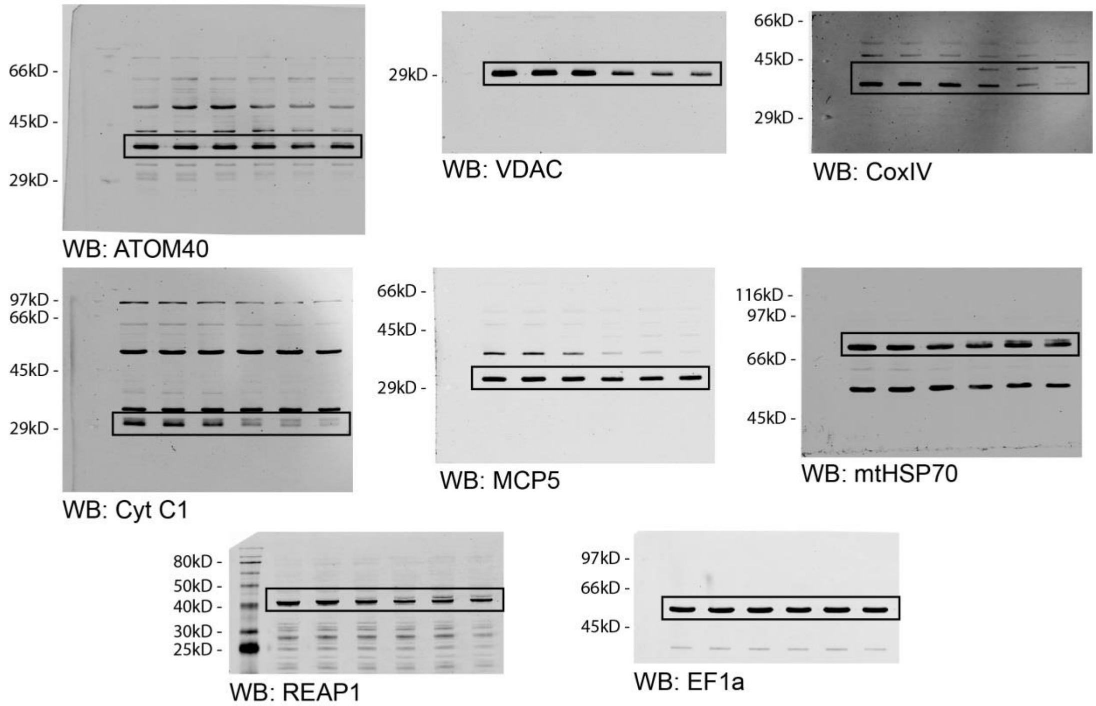

Figure 3a center panel:

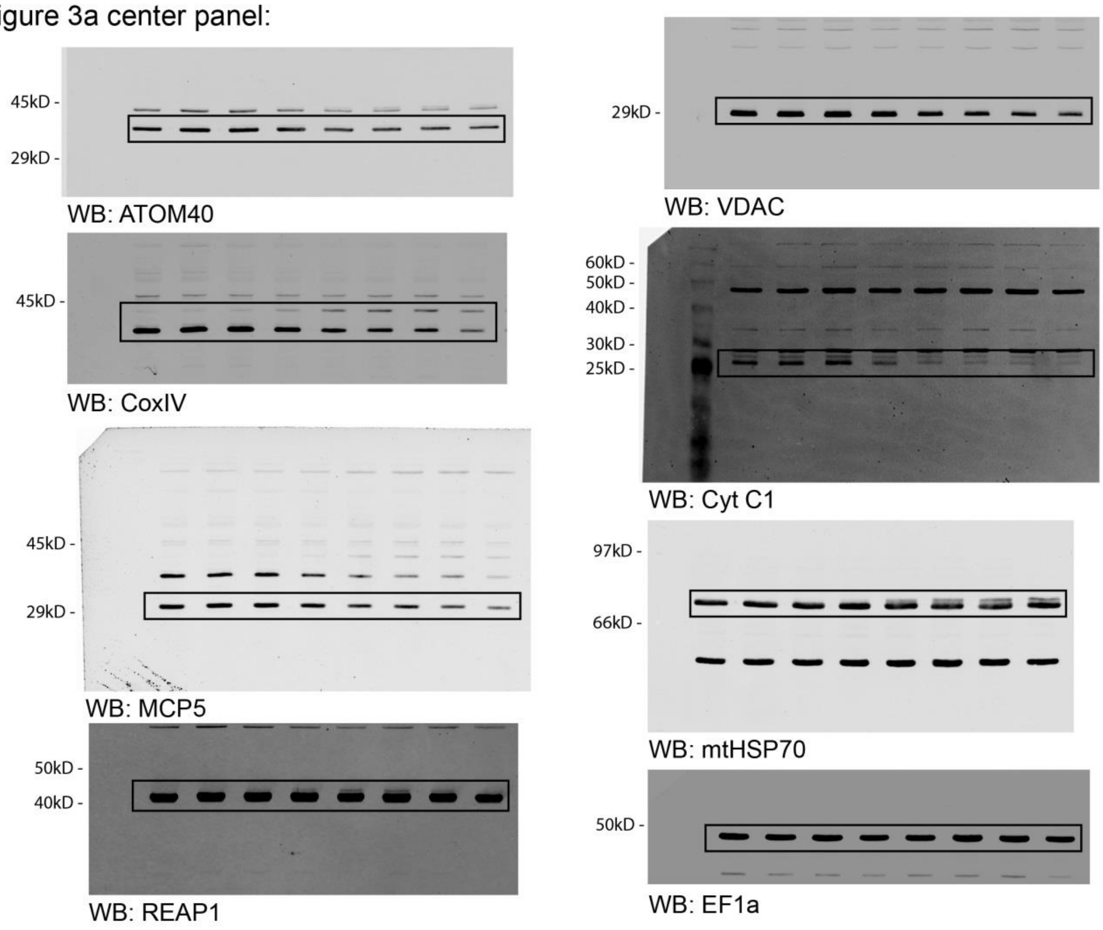

Figure 3a right panel:

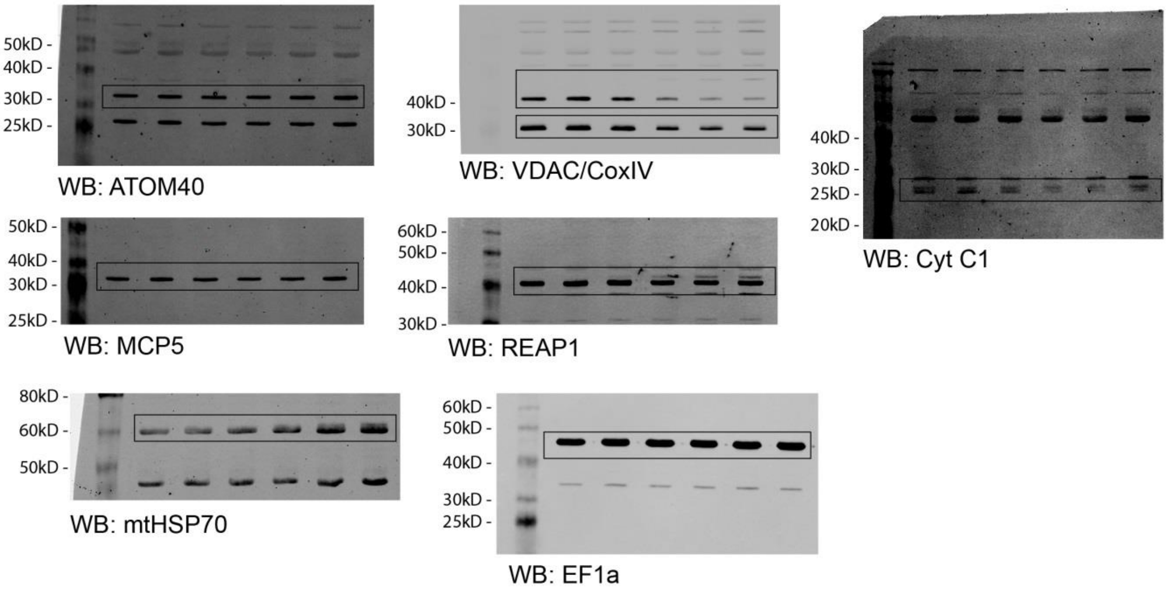

Figure 3b:

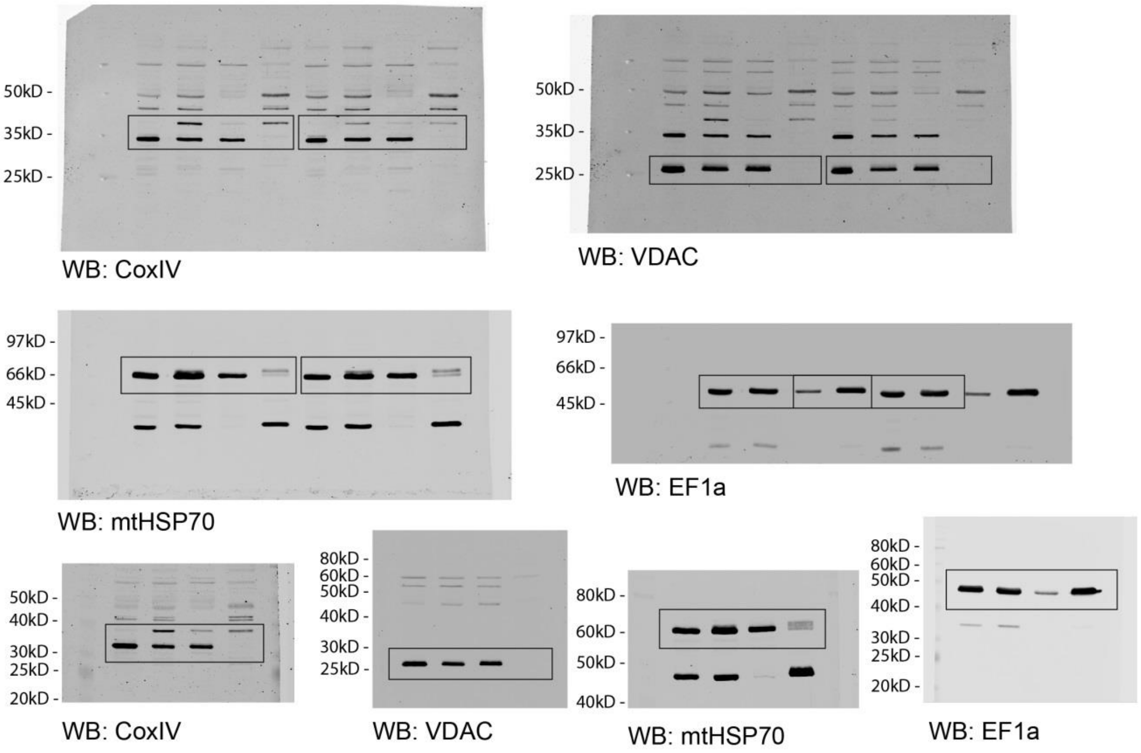

Figure 3c:

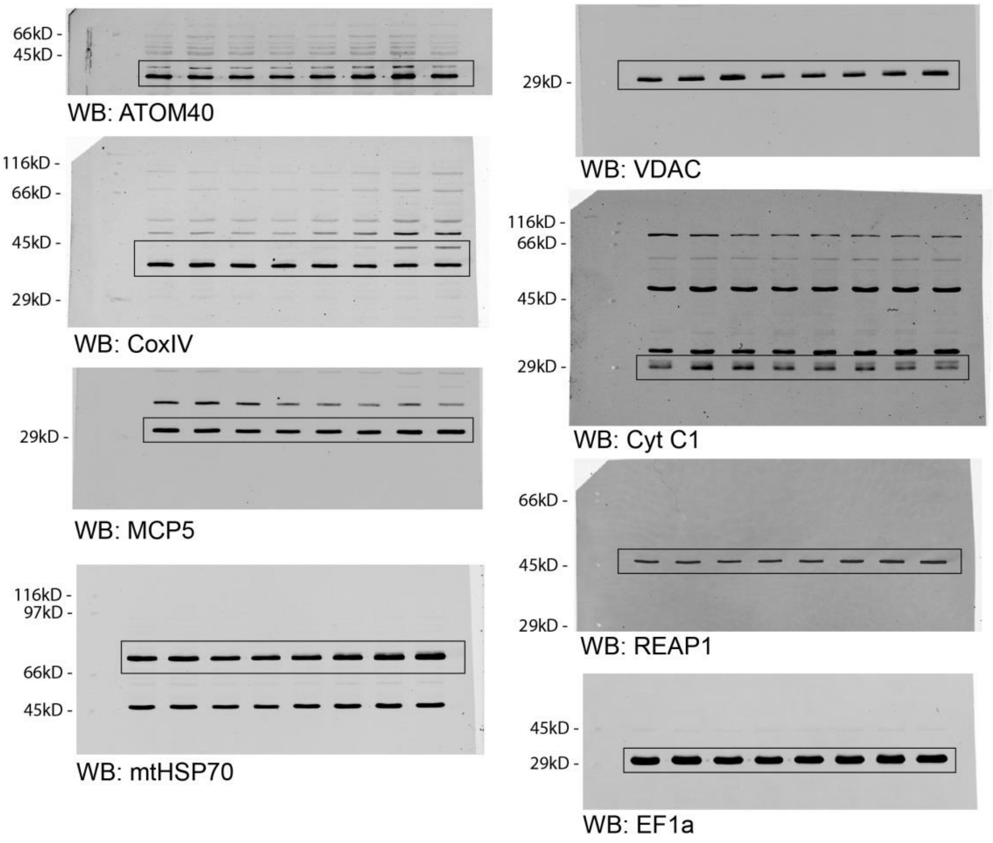

Figure 4:

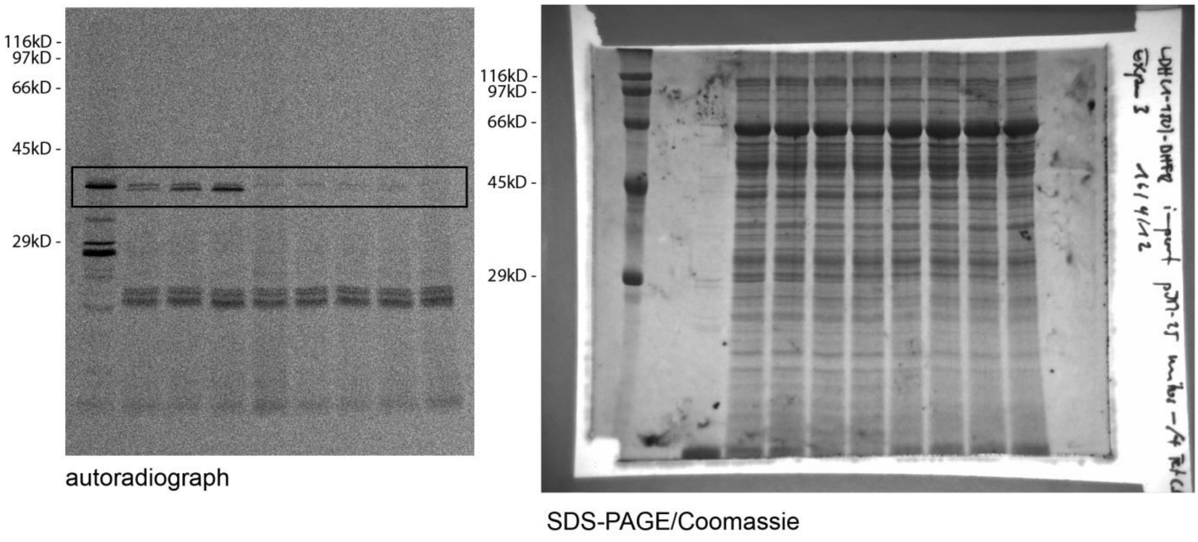

Figure 4 cont.:

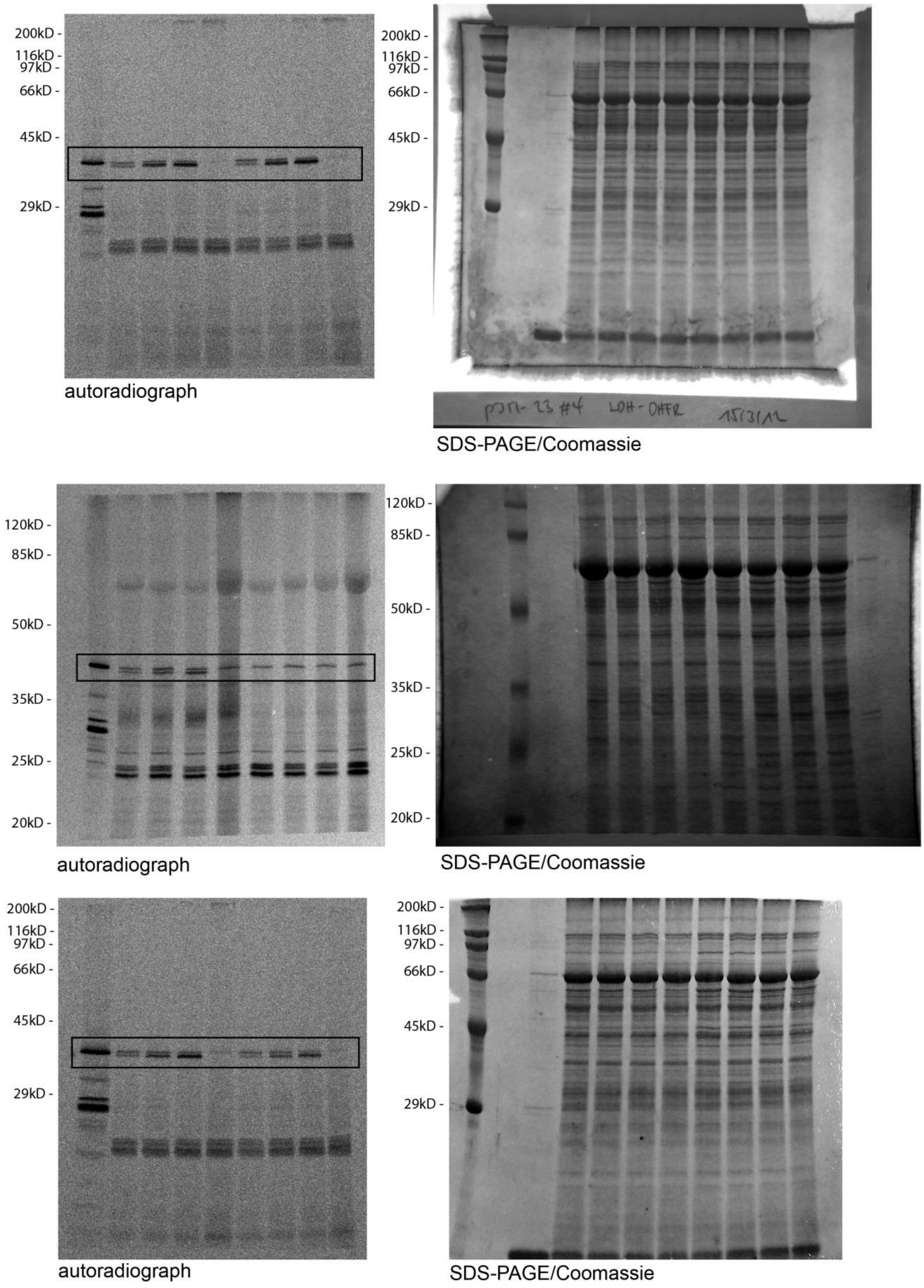

Figure 5a:

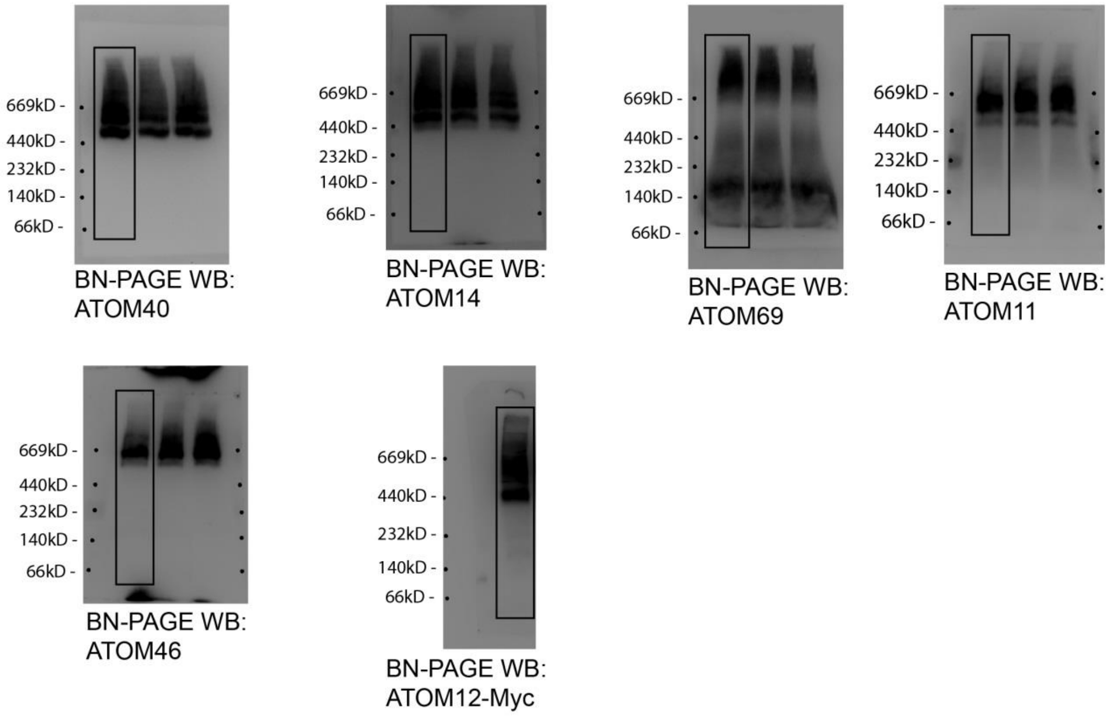

Figure 5c:

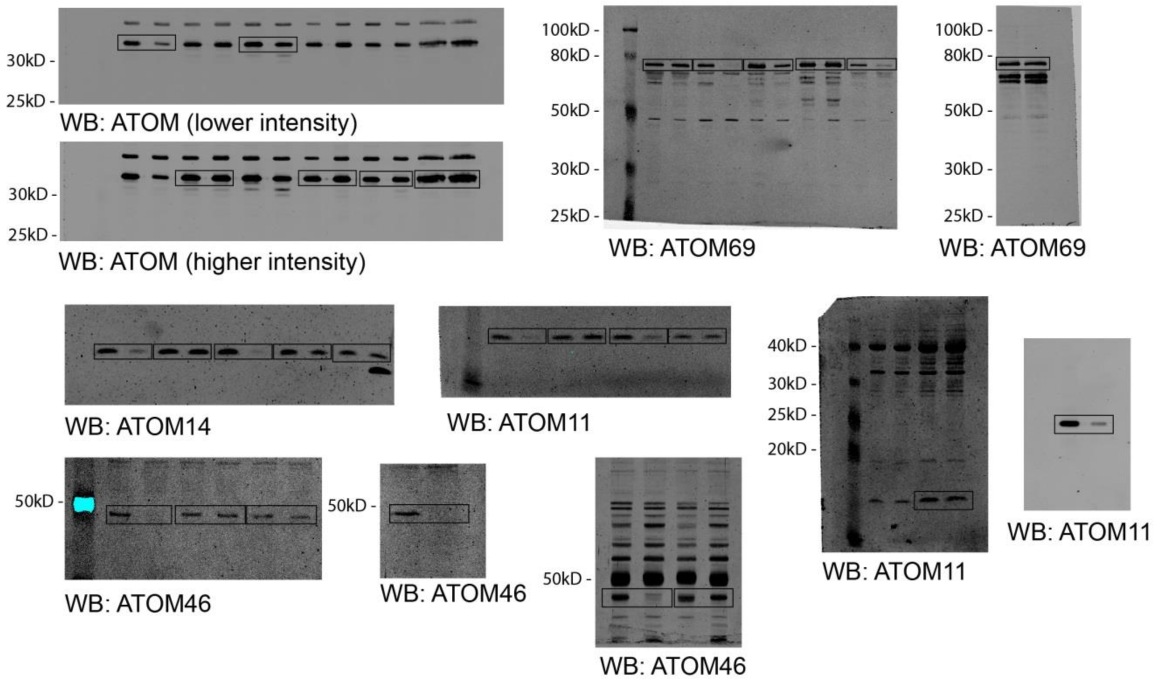

Figure 6b:

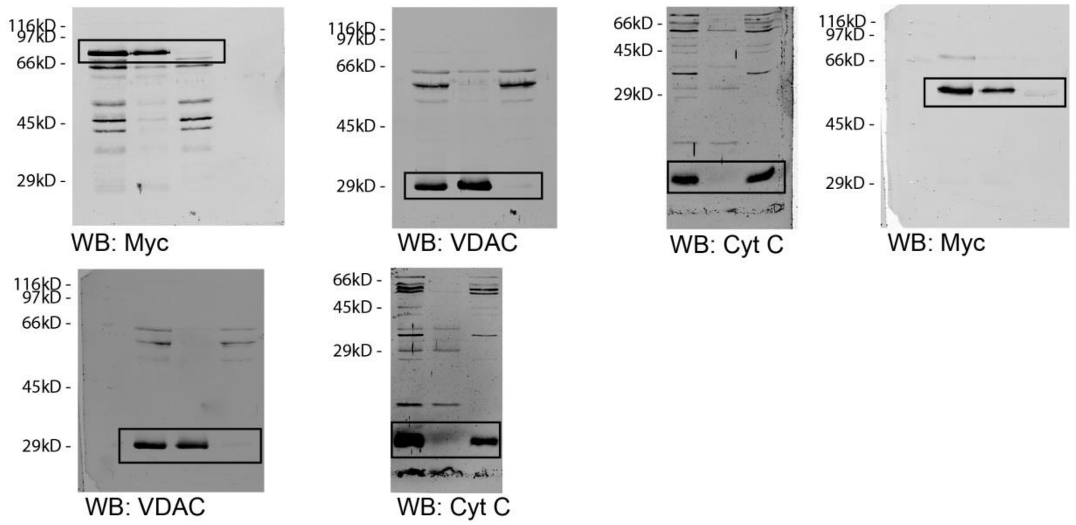

Figure 6c:

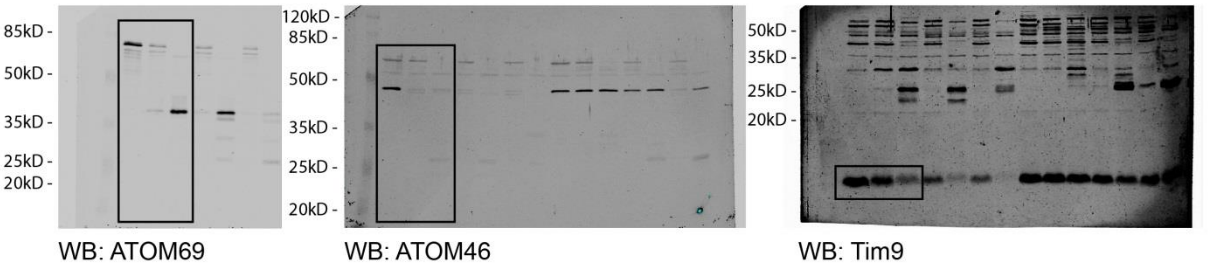

Figure 6d:

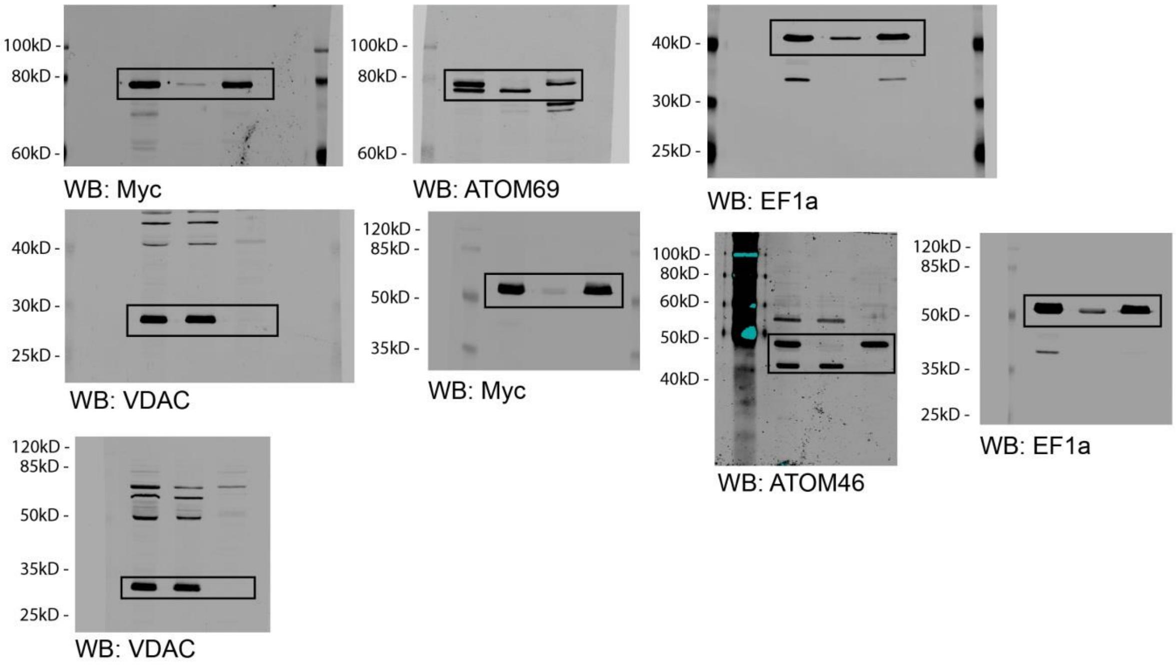

Figure 7a:

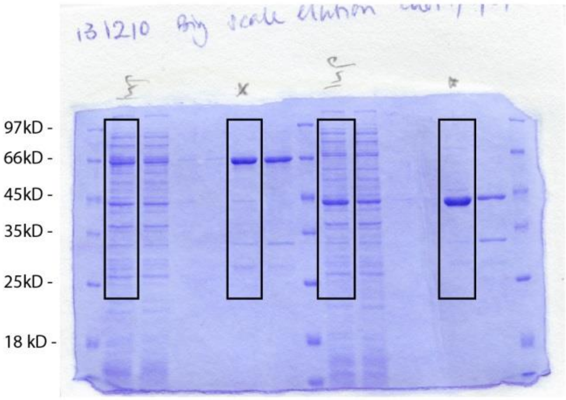

SDS-PAGE/Coomassie

Figure 7b:

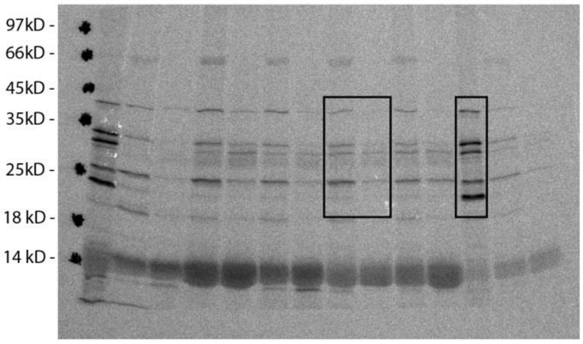

autoradiograph

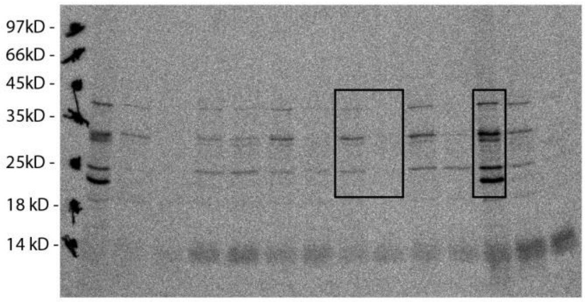

autoradiograph

Figure 7c:

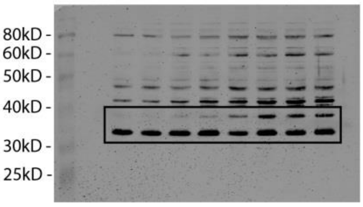

WB: CoxIV

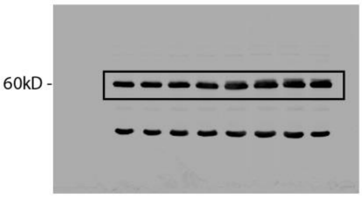

WB: mtHSP70

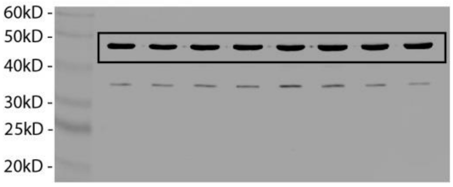

WB: EF1a

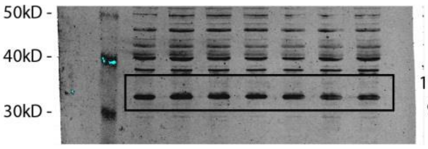

WB: CoxIV

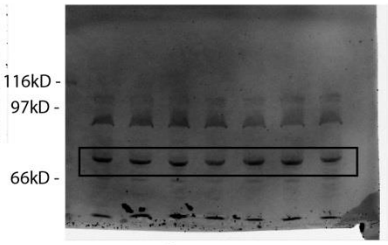

WB: mtHSP70

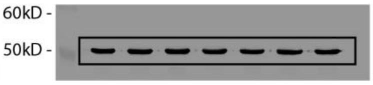

WB: EF1a

Figure 7d:

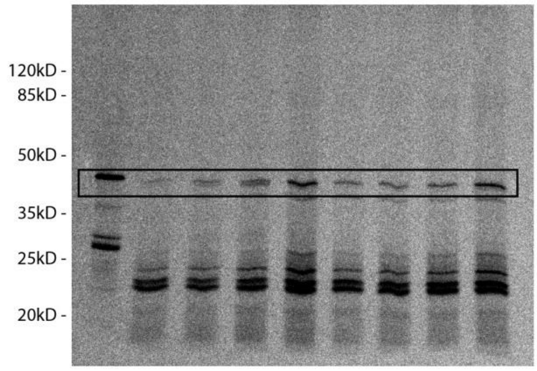

autoradiograph

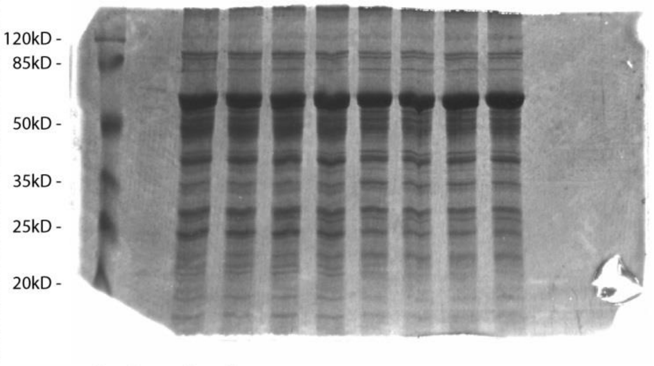

SDS-PAGE/Coomassie

Supplementary Figure 6. Full scans of blots, autoradiographs and gels presented in the paper.

	T. brucei	*T. cruzi*	*T. congolense*	*T. vivax*	*L. mexicana*	*L. braziliensis*	*L. major*
**ATOM40**	Tb927.9.9660	TcCLB.506885.340	TcIL3000_9_3480	TvY486_0904160	LmxM.34.4860	LbrM.34.4820	LmjF.35.4860
**ATOM14**	Tb927.11.5600	TcCLB.508647.110	TcIL3000.11.5900	TvY486_1105790	LmxM.24.0940	LbrM.24.0950	LmjF.24.0940
**ATOM69**	Tb927.11.11460	TcCLB.511803.40	TcIL3000_0_22040	TvY486_1112480	LmxM.28.2170	LbrM.28.2370	LmjF.28.2170
**ATOM46**	Tb927.11.7780	TcCLB.508813.60	TcIL3000.11.8270	TvY486_1108470	LmxM.28.0930	LbrM.28.1000	LmjF.28.0930
**ATOM11**	Tb927.10.11030	TcCLB.507711.300	TcIL3000_10_9240	TvY486_1010660	LmxM.32.0420	LbrM.33.0410	LmjF.33.0420
**ATOM12**	Tb927.8.4380	TcCLB.506865.50	TcIL3000_8_4110	TvY486_0803800	LmxM.10.0945	LbrM.10.1600	LmjF.10.0945

	Bodo saltans	*Strigomonas culicis*	*Angomonas deanei*	*C. fasciculata*	*Phytomonas EM1*	*Phytomonas HART1*
**ATOM40**	BS06545	EPY27979.1	EPY41482.1	CfaC1_31_5030	CCW64387.1	CCW68975.1
**ATOM14**	-	EPY31419.1	EPY39599.1	CfaC1_27_1010	CCW62531.1	CCW66035.1
**ATOM69**	BS62925	EPY27920.1	EPY28830.1	CfaC1_26_2850	CCW62280.1	CCW68618.1
**ATOM46**	BS59350	EPY36222.1	EPY38031.1	CfaC1_26_1180	CCW62376.1	CCW68713.1
**ATOM11**	BS82575	EPY29643.1	EPY25168.1	CfaC1_35_0690	CCW63059.1	CCW66153.1
**ATOM12**	BS29230	EPY15734.1	EPY40801.1	CfaC1_18_1760	CCW63569.1	CCW71368.1

Supplementary Table 1. ATOM complex subunits are highly conserved among kinetoplastid species. BLAST searches were performed with default search parameters using the *T. brucei* protein sequences of ATOM40, ATOM14, ATOM69, ATOM46, ATOM11 and ATOM12 as queries.

Description	Plasmid name	Primers	Remarks
pLew100-based C-terminal 3X c-Myc tagging vector	pJM1	TA**AAGCTT**GCG**ACCGGT**GCC**GGATCC**GGTACCGGGCCCCCCCTCG	pMOTag33M[1] served as a PCR template. The PCR product was cloned into a pLEW100-derived expression vector[2]
		GATCTCGGAGGAGGATCTGCTGTG**AGATCT**TG	
pLew100-based N-terminal 3X c-Myc tagging vector	pJM2	TA**GATATC**ATGGAGGAGCAGAAGCTGATCTCGGAGGAGGATCTGCTGAGATCCGAGGAGCAGAAGCTGATCTCGGAAGAAGACCTTCTTCGCTCGGAA	Primers were annealed and used for primer extension. The product was cloned into a pLEW100-derived expression vector[2]
		CA**GGATCC**GGC**ACCGGT**CGC**AAGCTT**CTCGAGGGGGGGCCCGGTACCCAGATCCTCCTCCGAGATCAGCTTCTGCTCTTCCGAGCGAAGAAGGTCTTCTTC	
pLew100-based TEV C-terminal 3X c-Myc tagging vector	pJM73	GATCCGAAAATTTATATTTTCAAGGTA	Primers were annealed and cloned into pJM1
		GATCTACCTTGAAAATATAAATTTTCG	
pLew100-based N-terminal 3X c-Myc TEV tagging vector	pJM74	AGCTGGAAAATTTATATTTTCAAGGTA	Primers were annealed and cloned into pJM2
		CCTTTTAAATATAAAAGTTCCATTCGA	
ATOM40 3X HA *in situ* tagging	-	CGCAGTGGTTTCAACCAGTTTATGTGTATGTACAATGCGTTGTACGACATTCACTCAGGAGGTTTGAAACATGGTGTGGTATTCACTGCCGGTACCGGGCCCCCCCTCGAG	pMOTag3H[1] or pFT22, a blasticidine resistance carrying derivative of pMOTag3H[1], was used as a PCR template
		ACACGACACGCCAATCGCTACTTCCTTTCCCATGTTTGCTCTTAAATTCCCTAACCTTTTCGTCCGCTGGGCAAAGACACACCATAACTTTGGCGGCCGCTCTAGAACTAGTGGAT	
ATOM14-Myc	pJM4	TA**AAGCTT**ATGACTGAAGCACTCTACAAC	427 gDNA served as PCR template. PCR product was cloned into pJM1
		CA**GGATCC**AATGCCATGACGTTCACGATAC	
Myc-ATOM14	pJM17	TA**AAGCTT**ATGACTGAAGCACTCTACAAC	427 gDNA served as PCR template. PCR product was cloned into pJM2
		CA**GGATCC**TTAAATGCCATGACGTTCACG	
ATOM69-Myc	pJM12	TA**AAGCTT**ATGTCTAGTGACGCCACTGCTG	427 gDNA served as PCR template. PCR product was cloned into pJM1
		CA**GGATCC**GAACTGGAGAGGTTGTGAGGC	
Myc-ATOM69	pJM13	TA**AAGCTT**ATGTCTAGTGACGCCACTGCTG	427 gDNA served as PCR template. PCR product was cloned into pJM2
		CA**GGATCC**TCAGAACTGGAGAGGTTGTGAG	
ATOM11-Myc	pJM9	TA**AAGCTT**ATGATGTTCGGTCGCCC	427 gDNA served as PCR template. PCR product was cloned into pJM1
		CA**GGATCC**GAATTCCTCTTCGTCCTCC	
Myc-ATOM11	pJM18	TA**AAGCTT**ATGATGTTCGGTCGCCC	427 gDNA served as PCR template. PCR product was cloned into pJM2
		CA**GGATCC**CTAGAATTCCTCTTCGTCCTC	
ATOM12-Myc	pJM6	TA**AAGCTT**ATGTTTATGATGCTCAACGG	427 gDNA served as PCR template. PCR product was cloned into pJM1
		CA**GGATCC**AGCAGTGGAGGAAACATAC	
Myc-ATOM12	pJM21	TA**AAGCTT**ATGTTTATGATGCTCAACGG	427 gDNA served as PCR template. PCR product was cloned into pJM2
		CA**GGATCC**TCAAGCAGTGGAGGAAACATAC	
ATOM46-Myc	pJM3	TA**AAGCTT**ATGGAGTCGCCGATCAACC	427 gDNA served as PCR template. PCR product was cloned into pJM1
		CA**GGATCC**CATGCCGCCACCCATCATTG	
POMP10-Myc	pJM8	TA**AAGCTT**ATGCCTTCAAAGCGGAAAAC	427 gDNA served as PCR template. PCR product was cloned into pJM1
		CA**ACCGGT**GATGAACTCGAGGGACTCC	
ATOM14 RNAi	pJM25	TCG**AAGCTTGGATCC**ATGACTGAAGCACTCTACAAC	pJM17 served as PCR template. PCR product was cloned into a pLEW100-derived stemloop RNAi vector[3]
		CA**TCTAGACTCGAG**TTAAATGCCATGACGTTCACG	
ATOM69 RNAi	pJM23	TCG**AAGCTTGGATCC**GCAGAAGACGATTCTGGAGG	pJM12 served as PCR template. PCR product was cloned into a pLEW100-derived stemloop RNAi vector[3]
		CA**TCTAGACTCGAG**GTCAAATTGTTCGGCAACCT	
ATOM12 RNAi	pTDN11	CCC**AAGCTTGGATCC**CTCAACGGTACCGCTGACGG	427 gDNA served as PCR template. PCR product was cloned into a pLEW100-derived stemloop RNAi vector[3]
		GC**TCTAGACTCGAG**GCAGTGGAGGAAACATACTCCC	
ATOM46 RNAi	pJM30	TCG**AAGCTTGGATCC**GGTATGACTCGCCTCTCTGC	pJM3 served as PCR template. PCR product was cloned into a pLEW100-derived stemloop RNAi vector[3]
		CA**TCTAGACTCGAG**CGCGTAGTGCACAGACATTT	
ATOM 3' UTR RNAi	pJM54	AGTCG**AAGCTTGGATCC**GCGGCGTAGAAAAAGTGAAC	427 gDNA served as PCR template. PCR product was cloned into a pLEW100-derived stemloop RNAi vector[3]
		CGTCA**TCTAGACTCGAG**AACGGGCTCGTTTATGAGAA	
ATOM11 conditional knockout	-	ATACAGTTTCTGCCCTTTGTAACAATCTTCTTCCTTGTGTGCTTGTGTAACAGACTACCTTCTCGTTAGTGTAACAAGTCCTCTGCAGTGATGGCCAAGTTGACCAGTGCCGTTCCG	Primers used for PCR of phleomycine resistance gene from pMOTag5[1]
		CTTCTTGCGTTGCCAAATCGTCCGCCTTCTCAGTTGCATGTTAACCGACGTTCACTTTTTCTACGCCGCCAAGCCCTACGACAAACGGCTTCAGTCCTGCTCCTCGGCCACGAAGTG	
		ATACAGTTTCTGCCCTTTGTAACAATCTTCTTCCTTGTGTGCTTGTGTAACAGACTACCTTCTCGTTAGTGTAACAAGTCCTCTGCAGTGATGGCCAAGCCTTTGTCTCAAGAAG	Primers used for PCR of blasticidine resistance gene from a modified version of pMOTag3H[1]
		CTTCTTGCGTTGCCAAATCGTCCGCCTTCTCAGTTGCATGTTAACCGACGTTCACTTTTTCTACGCCGCCAAGCCCTACGACAAACGGCTTTAGCCCTCCCACACATAACCAGAG	
		GGCAACCTGGGAAGAGAAGT	Primers used to confirm successfull knockout of ATOM11
		TTGCATGTTAACCGACGTTC	
		GGAGCGGTCGAGTTCTGG	
		ATCAACAGCATCCCCATCTC	
		GGAGCCCTACAACTCTGTGC	alpha-tubulin specific primers to check integrity of gDNA
		CACCCTCTTCCATACCCTCA	
ATOM69-ΔTMH	pJM77	TGCTG**AAGCTTATG**TCTAGTGACGCCACTGCTG	pJM13 served as a PCR template. PCR product was cloned into pJM73
		CAGCA**GGATCC**TTGATTGCCCTTCTTTAGTCTCCC	
ΔTMH-ATOM46	pJM78	TGCTG**AAGCTT**ATGCTATCATCACTGGATGCTGAC	pJM3 served as PCR template. PCR product was cloned into pJM74
		CAGCA**GGATCC**TTACATGCCGCCACCCATCAT	
ATOM69-ΔTMH-His_6	pSD10	ATGAG**CATATG**TCTAGTGACGCCACTGC	427 gDNA served as PCR template. PCR product was cloned into pET21a(+) (Novagen)
		CACGA**AAGCTT**TGCACCCGACCTTTGATTG	
His_6-ΔTMH-ATOM46	pSD9	ATGAG**CATATG**CTATCATCACTGGATGCTG	427 gDNA served as PCR template. PCR product was cloned into pET28a(+) (Novagen)
		CACGA**AAGCTT**TTACATGCCGCCACCCATC	

Supplementary Table 2. List of all primers used for cloning procedures and establishment of cell lines.

Supplementary references

1 Oberholzer, M., Morand, S., Kunz, S. & Seebeck, T. A vector series for rapid PCR-mediated C-terminal in situ tagging of *Trypanosoma brucei* genes. *Mol. Biochem. Parasitol.* **145**, 117-120 (2005).

2 Wirtz, E., Leal, S., Ochatt, C. & Cross, G. A. A tightly regulated inducible expression system for conditional gene knock-outs and dominant-negative genetics in *Trypanosoma brucei*. *Mol. Biochem. Parasitol.* **99**, 89-101 (1999).

3 Bochud-Allemann, N. & Schneider, A. Mitochondrial substrate level phosphorylation is essential for growth of procyclic *Trypanosoma brucei*. *J. Biol. Chem.* **277**, 32849-32854 (2002).
